# *Bifidobacterium animalis* subsp. *lactis* A6 ameliorates bone and muscle loss via modulating gut microbiota composition and enhancing butyrate production

**DOI:** 10.1038/s41413-024-00381-1

**Published:** 2025-02-25

**Authors:** Ming Chen, Yi Li, Zhengyuan Zhai, Hui Wang, Yuan Lin, Feifan Chang, Siliang Ge, Xinyu Sun, Wei Wei, Duanyang Wang, Mingming Zhang, Ruijing Chen, Haikuan Yu, Taojin Feng, Xiang Huang, Dongliang Cheng, Jiang Liu, Wenxuan Di, Yanling Hao, Pengbin Yin, Peifu Tang

**Affiliations:** 1 Senior Department of Orthopedics, The Fourth Medical Center of Chinese PLA General Hospital, Beijing, China; 2 National Clinical Research Center for Orthopedics, Sports Medicine & Rehabilitation, Beijing, China; 3 Key Laboratory of Precision Nutrition and Food Quality, Department of Nutrition and Health, China Agricultural University, Beijing, China; 4 The Department of Orthopedic Surgery, Second Affiliated Hospital of Harbin Medical University, Harbin, China; 5 Department of Clinical Nutrition, Peking Union Medical College Hospital, Chinese Academy of Medical Science and Peking Union Medical College, Beijing, China

**Keywords:** Bone, Osteoporosis

## Abstract

Systematic bone and muscle loss is a complex metabolic disease, which is frequently linked to gut dysfunction, yet its etiology and treatment remain elusive. While probiotics show promise in managing diseases through microbiome modulation, their therapeutic impact on gut dysfunction-induced bone and muscle loss remains to be elucidated. Employing dextran sulfate sodium (DSS)-induced gut dysfunction model and wide-spectrum antibiotics (ABX)-treated mice model, our study revealed that gut dysfunction instigates muscle and bone loss, accompanied by microbial imbalances. Importantly, *Bifidobacterium animalis* subsp. *lactis* A6 (*B. lactis* A6) administration significantly ameliorated muscle and bone loss by modulating gut microbiota composition and enhancing butyrate-producing bacteria. This intervention effectively restored depleted butyrate levels in serum, muscle, and bone tissues caused by gut dysfunction. Furthermore, butyrate supplementation mitigated musculoskeletal loss by repairing the damaged intestinal barrier and enriching beneficial butyrate-producing bacteria. Importantly, butyrate inhibited the NF-κB pathway activation, and reduced the secretion of corresponding inflammatory factors in T cells. Our study highlights the critical role of dysbiosis in gut dysfunction-induced musculoskeletal loss and underscores the therapeutic potential of *B. lactis* A6. These discoveries offer new microbiome directions for translational and clinical research, providing promising strategies for preventing and managing musculoskeletal diseases.

## Introduction

Osteosarcopenia, characterized by systematic muscle and bone loss, is a complex metabolic disorder often linked to gut dysfunction.^1–3^ Chronic gastrointestinal diseases, particularly inflammatory bowel diseases (IBDs), exemplify this association and have emerged as a global health concern.^4^ Notably, over one-third of IBD patients undergo muscle and bone loss, drastically enhancing their risk of falls, fractures, vulnerability, and mortality.^2,5^ Currently, pharmaceutical interventions for sarcopenia are still under development, while osteoporosis treatments come with undesirable side effects such as jaw necrosis and atypical fractures.^6,7^ Moreover, our understanding of skeletal changes in individuals with gut dysfunction remains limited, highlighting the urgent need for in-depth research in this area.

The gut is a dynamic microecosystem continuously interacting with various extraintestinal organs, playing a crucial role in maintaining physiological health.^8–10^ These interactions underscore the gut’s pivotal role in addressing bone and muscle loss. The research proposes the gut-musculoskeletal axis theory and suggests that intestinal microorganisms influence muscle and bone function.^11–13^ For instance, Lahiri et al. demonstrated that mice lacking typical microbiome exhibited muscle atrophy and reduced skeletal function, with these issues reversed by transplanting microbiota from pathogen-free mice.^14^ Although prior studies have observed muscle^15^ or bone^16^ loss in IBD mouse models, the mechanisms by which gut changes influence the progression of bone and muscle loss remain unclear. Consequently, directing interventions toward gut may offer viable strategies to counteract musculoskeletal deterioration.

Probiotics, known for promoting beneficial gut microbes, show promise in enhancing physical fitness. These live microorganisms have demonstrated effectiveness in mitigating various health issues, including diarrhea, respiratory distress, and functional constipation.^17^ Recent findings, such as those by Zhu et al., have highlighted the positive effects of probiotics on kidney diseases, particularly *Lactobacillus casei* Zhang’s ability to rectify dysbiosis and alleviate kidney injury.^18^ Although earlier studies hinted at the impact of probiotic treatment on bone and muscle health, the evidence remained inconclusive due to study limitations and population variability.^19–21^ Therefore, there is a growing interest in exploring the potential of specific probiotics in protecting against bone and muscle loss.

In this study, we found that exposure to dextran sulfate sodium (DSS) not only induced bone and muscle loss in mice but also disrupted gut microbiota balance. Crucially, our results demonstrate that a probiotic, *Bifidobacterium animalis* subsp. *lactis* A6 (*B. lactis* A6), can mitigate muscle and bone loss. This occurs through the modification of the intestinal microbial balance, the promotion of butyrate-producing bacteria, and the restoration of depleted butyrate levels in serum, muscle, and bone induced by DSS exposure. Furthermore, butyrate supplementation effectively alleviates muscle and bone loss by repairing the damaged intestinal barrier and enriching beneficial butyrate-producing bacteria. Importantly, butyrate inhibited the NF-κB pathway activation and reduced the secretion of corresponding inflammatory factors in T cells, contributing to its synergistic protective effects on musculoskeletal tissues. These results offer valuable insights into the role of *B. lactis* A6 in mitigating bone and muscle loss related to gut dysfunction, which can inform translational and clinical recommendations for probiotic interventions targeting gastrointestinal and musculoskeletal disorders.

## Results

### DSS-induced gut dysfunction leads to bone and muscle loss in mice

**Fig. 1:**
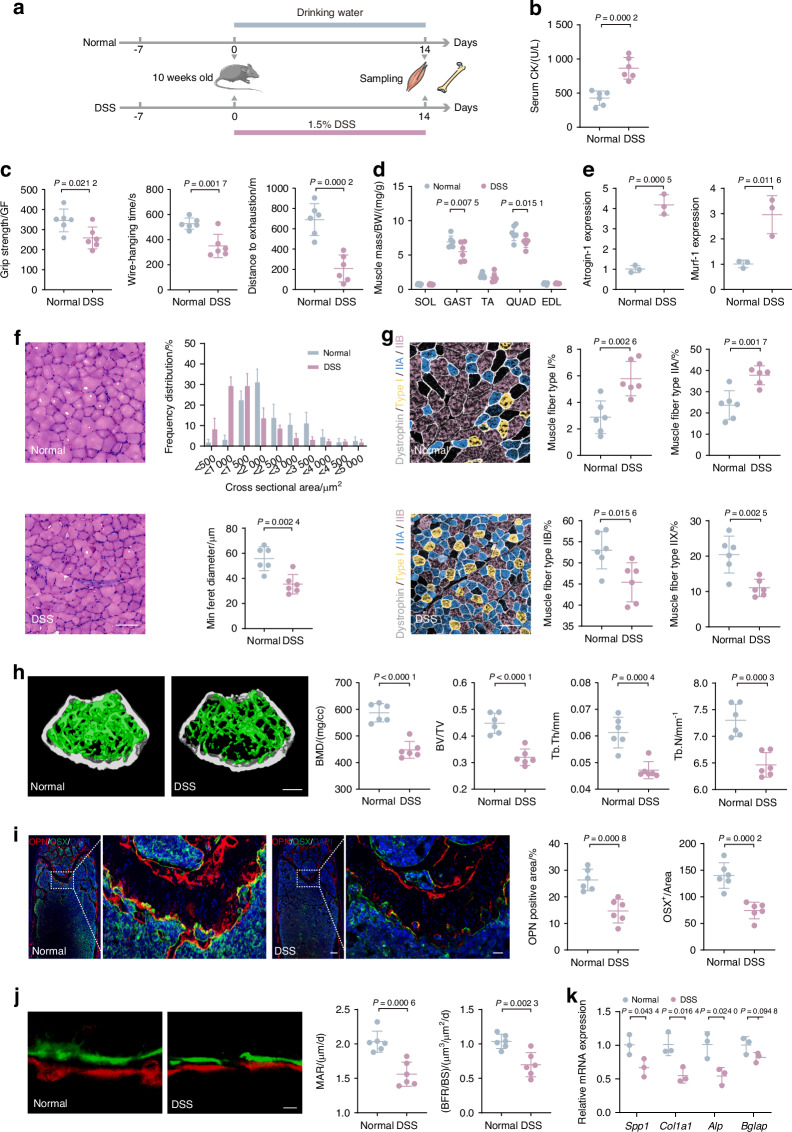
DSS-induced gut dysfunction leads to bone and muscle loss in mice. **a** Schematic representation illustrating the experimental design. Mice were exposed to dextran sodium sulfate (DSS) in DSS group and PBS in normal group for 2 weeks. **b** Measurement of CK activity of the serum (*n* = 6). **c** Assessment of physical performance using all-limb force, longest suspension time, and distance to exhaustion evaluated by handgrip, hanging wire tests, and treadmill, respectively (*n* = 6). **d** Muscle mass analysis from each group (*n* = 6). **e** mRNA expressions of *Atrogin-1* and *Murf-1* in gastrocnemius, tested by qPCR (*n* = 3). **f** Representative images of H&E staining in gastrocnemius cross-sections, with the frequency distribution of CSA and quantification of average minimal Feret’s diameter of myofibers (*n* = 6). Scale bar, 100 μm. **g** Representative immunofluorescence images to visualize specific types of muscle fibers and quantification. Type I (purple), type IIA (green), type IIX (not shown), and type IIB (red) (*n* = 6). Scale bar, 100 μm. **h** Representative micro-CT images of distal femoral metaphyseal trabecular bone. Quantitative analysis of bone mass, including BMD, BV/TV, Tb. Th, and Tb. N (*n* = 6). Scale bar, 500 μm. **i** Representative images of OSX (green) and OPN (red) immunostainings and quantification of OPN^+^ and OSX^+^ area on distal femurs (*n* = 6). Scale bar, 200 and 50 μm, respectively. **j** Representative images and quantification of new bone formation assessed by dynamic histomorphometric analyses (*n* = 6). Scale bar, 25 μm. **k** mRNA Expressions of osteogenesis gene (*Spp1*, *Col1a1*, *Alp*, and *Bglap*) expression in tibia, tested by qPCR (*n* = 3). Values are represented as the average ± standard deviation. The significance level (*P* value) was determined through a two-sided Welch’s *t*-test

To investigate the connection between gut dysfunction and musculoskeletal deterioration, we induced colitis in mice with DSS exposure (Fig. 1a). The phenotypes of the gut were assessed, revealing disrupted intestinal function (Fig. S1). Serum creatine kinase (CK) concentrations have historically been used as muscle damage indicator,^22^ and our finding that DSS-exposed mice had increased serum CK levels indicated apparent muscle damage (Fig. 1b). Subsequent behavioral analyses revealed that DSS-exposed mice displayed significant reductions in limb grip strength, wire-hanging time, and exercise capacity (Fig. 1c). Thus, gut dysfunction does deleteriously affect muscle function in mice. Through dissecting the posterior limbs of mice, gastrocnemius (Gast) and quadriceps (Quad) weights were found significantly reduced (Fig. 1d); these altered muscle profiles were consistent with the changes we observed in behavior analysis. Moreover, qPCR analysis showed increased expression of muscle atrophy markers *Atrogin-1* and *Murf-1*, both of which encode E3 ubiquitin ligases,^23^ in Gast after DSS exposure (Fig. 1e).

To investigate the effects of DSS exposure on muscle structure, we performed H&E staining in Gast, which is widely recognized as a representative skeletal muscle due to its size, accessibility, and its ability to reflect the systemic skeletal muscle response.^24^ Muscle fibers of the DSS-exposed group displayed irregular shapes and sarcolemma membrane disruption. Besides, there was a leftward shift in the curve that represents the cross-sectional area (CSA), resulting in a marked decrease in Feret’s diameter (Fig. 1f). Different fiber types have unique physiological and metabolic properties, and the fiber type compositions can change under conditions like aging, injury, and physical training, which potentially affects muscle functions.^25,26^ Immunofluorescence staining for fiber types revealed that in typical mice, type IIB fibers predominated, succeeded by types IIA, IIX, and I (Fig. S2a). However, DSS exposure led to noticeable reductions in the proportion of type IIB and IIX fibers and increases in type I and IIA fibers, suggesting a transition from the glycolytic nature toward the oxidative characteristics (Fig. 1g and Fig. S2a). These findings suggest a shift in muscle metabolism after DSS exposure, which may have contributed to the observed muscle function declines.

Additionally, microcomputed tomography (micro-CT) scans showed decreased trabecular bone in the femur of DSS-exposed mice, reflected by reduced bone mineral density (BMD), bone volume/total volume (BV/TV), trabecular thickness (Tb. Th) and number of trabeculae (Tb. N) (Fig. 1h). These findings confirmed that the DSS exposure resulted in reduced bone mass and compromised bone microarchitecture. Mechanical stress testing further revealed significant decreases in maximum load, energy to ultimate load, and breaking energy in the DSS-exposed mice (Fig. S2b), indicating mechanical defects and bone fragility. Osteoblasts are essential for bone formation.^27^ To study osteogenic protein expression within the bone tissue, we performed immunofluorescence staining of femur samples target for bone osterix (OSX), osteopontin (OPN), osteocalcin (OCN), and tartrate-resistant acid phosphatase staining (TRAP).^28^ We found that DSS exposure decreased the numbers of OSX, OPN, and OCN (Fig. 1i and Fig. S2c), suggesting reduced bone-creating processes and mineral integration. We also found that TRAP staining enhanced in DSS-induced group (Fig. S2d), suggesting increased bone absorption. Moreover, we quantify bone metabolism markers in serum, which show the same result as immunofluorescence staining. The result showed that type I N-terminal propeptide (P1NP) as an indicator of bone formation decreased and C-terminal telopeptide of type I collagen (CTX-1) as a marker of bone resorption increased in serum (Fig. S2e). Additionally, as indicated by bone histomorphometric analyses, the mineral apposition rate (MAR) and bone formation rate (BFR) of the DSS-exposed group were all significantly reduced, providing further evidence of impaired bone formation (Fig. 1j). qPCR analysis of femur revealed decreased expression of osteogenesis-related genes, including secreted phosphoprotein 1 (*Spp1*), collagen type I alpha 1 chain (*Col1a1*), alkaline phosphatase (*Alp*), and bone gamma-carboxyglutamate protein (*Bglap*) (Fig. 1k). These findings collectively support that DSS exposure negatively impacts osteoblast physiology and bone formation.

The variance in microbiota composition across pathological conditions like DSS-induced colitis and different ages presents a compelling avenue for investigating its impact on bone and skeletal muscle mass and quality. Thus, we managed to evaluate the influence of microbiota from distinct sources on bone and skeletal muscle mass and quality in DSS-induced colitis mice. Our study employed a fecal microbiota transplantation (FMT) approach, wherein DSS-model mice were assigned into four intervention groups (Fig. S3a). We aim to dissect the differential impacts of microbiota derived from varied host conditions—specifically focusing on age-related and DSS-induced alterations in microbiota composition—on the pathophysiology of bone and skeletal muscle. Interestingly, after administrating FMT to mice, we observed notable reductions in serum CK levels in Normal-FMT and Young-FMT groups than DSS-FMT and Aged-FMT groups (Fig. S3b). In addition, behavioral analyses revealed Normal-FMT and Young-FMT groups have better muscle functions and larger Gast mass (Fig. S3c, d). Besides, H&E staining confirmed augmented muscle integrity and increased muscle fiber size in Normal-FMT and Young-FMT mice (Fig. S3e). For bone, micro-CT data showed enhanced skeletal density and structure with higher BMD in mice with Normal-FMT and Young-FMT (Fig. S3f). Moreover, Normal-FMT and Young-FMT mice showed a significant rise in quantitative enumeration of osteoblasts expressing OCN (Fig. S3g) and a decrease in the number of TRAP-positive osteoclasts compared with DSS-FMT and Aged-FMT group (Fig. S3h). These findings elucidate the differential impacts of microbiota from varied sources on bone and skeletal muscle mass and quality. What’s more, these results underscore the therapeutic potential of FMT, particularly from healthy and young donors, in mitigating the deleterious effects of DSS-induced colitis on bone and skeletal muscle health. These findings pave the way for further exploration of microbiota-based interventions in the management of musculoskeletal disorders associated with inflammatory conditions.

### Microbial dysbiosis characterizes gut dysfunction-induced bone and muscle loss in both mice models and human individuals

**Fig. 2:**
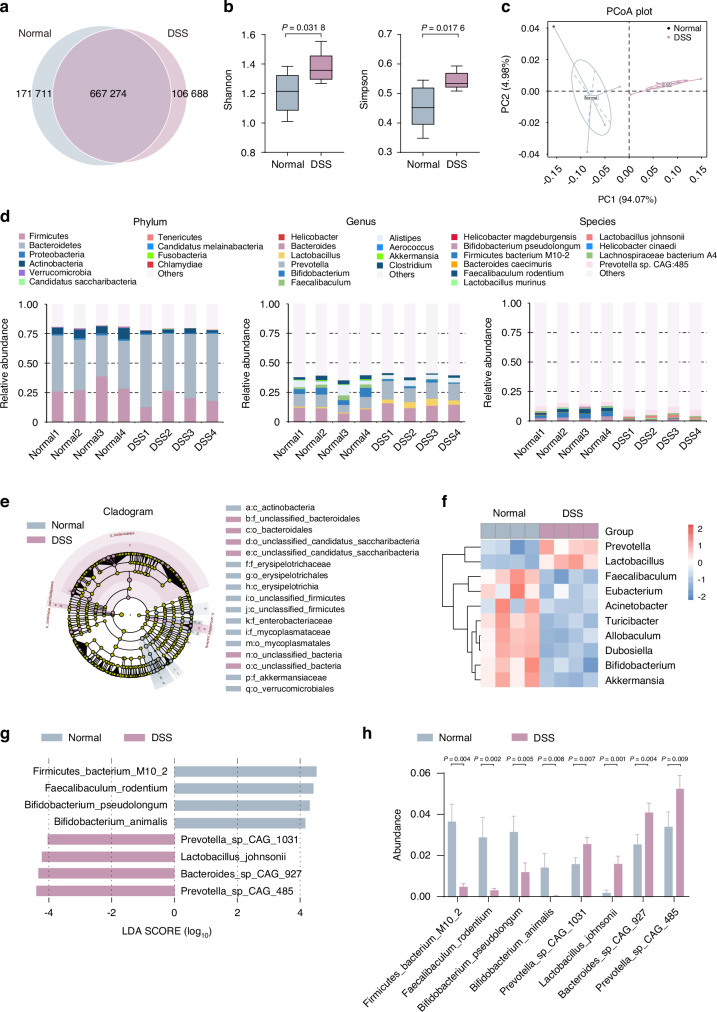
Metagenomic analysis reveals altered gut microbiota composition following DSS exposure. **a** Venn diagram analysis of gene numbers detected in two groups. **b** The box plot illustrates *α*-diversity using the Shannon and Simpson indices. **c** Principal coordinate analysis (PCoA) of *β*-diversity at the phylum tier is conducted via a Bray–Curtis matrix comparison for both groups. **d** Structure plot of the relative fecal bacterial abundances in phylum and genus-level based on Bray–Curtis distance. Analysis of cladogram generated from LEfSe (**e**) and the heatmap cluster (**f**) across different taxa levels. **g** Analysis of LDA score in the species level. **h** Quantitative analysis of differential taxa in two groups. Values are represented as the average ± standard deviation. The significance level (*P* value) was determined through a two-sided Welch’s *t*-test

To explore the gut microbiota’s role in bone and muscle loss, we analyzed microbiomes from control and DSS-treated mice. Through high-throughput Illumina sequencing, open reading frame (ORF) prediction was executed for scaftigs (Fig. S4a). The Venn diagram analysis was also performed (Fig. 2a), while species abundance comparisons were ascertained via Analyses of Similarity (Anosim) (Fig. S4b). Complementing these analyses, *α*-diversity (Shannon and Simpson indexes) (Fig. 2b) and *β*-diversity (principal coordinate analysis, PCoA) metrics (Fig. 2c) revealed significant microbial profile differences between these two groups. To further assess the gut microbiota changes, we performed subsequent taxonomic analysis extending from phylum to species level (Fig. 2d). At the phylum level, DSS exposure induced notable decreases in Firmicutes and Actinobacteria, and marked increases in Bacteroidetes (Fig. 2d, left). At the genus level, we observed decreased abundance of *Bifidobacterium*, *Akkermansia*, and *Clostridium*, while genera such as *Prevotella*, and *Lactobacillus* were increased (Fig. 2d, middle). At the species level, notable changes included decreased abundance of *Bifidobacterium pseudolongum* (*B. pseudolongum*) and *Faecalibaculum rodentium* (*F. rodentium*), and increased abundance of *Helicobacter magdeburgensis* and *Bacteroides caecimuris* after DSS exposure (Fig. 2d, right).

These shifts in microbiota composition were confirmed through cluster analysis of relative abundance (Fig. S4c) and LEfSe (Fig. 2e and Fig. S4d). Notably, there was lower abundance of *Bifidobacterium*, *Akkermansia*, *Eubacterium*, and *Faecalibaculum*, accompanied by higher presence of *Lactobacillus* and *Prevotella* in the DSS exposure group (Fig. 2e, f and Fig. S4c, d). Specifically, at the species level, the LDA score demonstrated reduced levels of *Firmicutes bacterium* M10-2, *F. rodentium*, *B. pseudolongum*, and *B. animalis*, with an increase in *Prevotella* sp CAG-1031, *Lactobacillus johnsonii*, *Bacteroides* sp CAG-927 and *Prevotella* sp CAG-485 after DSS exposure (Fig. 2g, h). Given *Bifidobacterium*’s well-documented health-promoting properties, including inflammation modulation, enhanced nutrient absorption, and reinforced gut barrier function,^29^ we explored how the decline of this probiotic strain might contribute to the pathogenesis of bone and muscle loss.

To further determine whether the changes in gut microbiota in colitis patients share any common characteristics with the changes in gut microbiota in the DSS-induced colitis mice, we collected samples from human IBD patients and healthy individuals, and performed microbiota analysis (Fig. S5). The Venn data analysis was performed to describe the gene numbers detected in IBD patients and health people (Fig. S5a). Augmenting the Venn data, the assessment of α-diversity, as manifested by the Shannon and Simpson indices (Fig. S5b), in conjunction with β-diversity, evaluated via PCoA metrics (Fig. S5c), underscored notable disparities in the microbial compositions between these two groups. To deepen the evaluation of alterations within the gut microbiota, a taxonomic analysis spanning from the phylum to the species level was performed (Fig. S5d). At the phylum level, IBD patients showed notable decreases in Firmicutes and Actinobacteria, and marked increases in Proteobacteria (Fig. S5d, left). At the genus level, there was a noted reduction in the populations of *Bifidobacterium*, *Treponema*, and *Blautia*, whereas an increase was observed in the genera *Escherichia*–*Shigella* and *Bacteroides* (Fig. S5d, middle). At the species level, notable changes included decreased abundance of *Blautia_sp_N6H1-15*, *Bifidobacterium_longum* and *Bifidobacterium_adolescenties*, and increased abundance of *Alistipes_puttredinis* and *Bacteroides_fragilis* in IBD patients (Fig. S5d, right). These shifts in microbiota composition were further confirmed through LEfSe and LDA scores (Fig. S5e, f). At the species level, the LDA score demonstrated that IBD patients showed reduced levels of *Bifidobacterium_adolescenties*, *Bifidobacterium_longum*, and Actinobacteria, with an increase in bacteria. Notably, there was lower abundance of Bifidobacteriaceae, Oscillospiraceae, and Prevotellaceae in IBD patients (Fig. S5e, f). Same as our result in DSS-induced colitis mice, we find the same decline of *Bifidobacterium* in colitis patients, thereby strengthening the common microbiota characteristics.

### Administration of probiotic *B. lactis* A6 alleviates bone and muscle loss

**Fig. 3:**
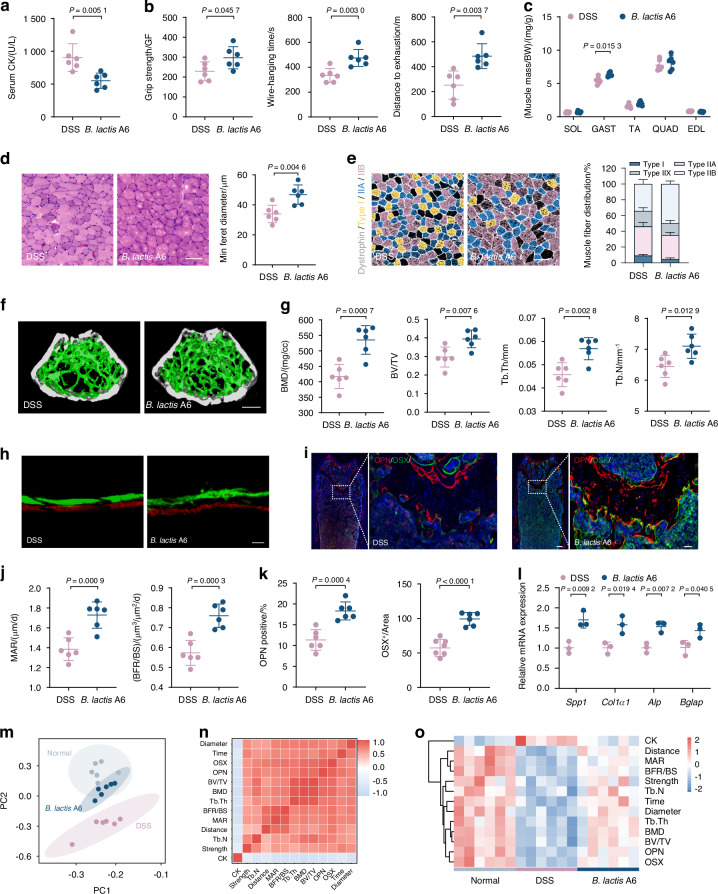
Administration of probiotic *Bifidobacterium animalis* subsp. *lactis* A6 (*B. lactis* A6) alleviates gut dysfunction-triggered bone and muscle loss. **a** Measurement of CK activity of the serum (*n* = 6). **b** Assessment of physical performance by handgrip, hanging wire tests, and treadmill, respectively (*n* = 6). **c** Muscle mass analysis from each group (*n* = 6). **d** Representative images of H&E staining in gastrocnemius cross-sections and quantification of average minimal Feret’s diameter of myofibers (*n* = 6). Scale bar, 100 μm. **e** Representative immunofluorescence images to visualize specific types of muscle fibers and fiber type compositions (*n* = 6). Scale bar, 100 μm. **f** Representative micro-CT images of distal femoral metaphyseal bone. **g** Quantitative analysis of bone mass (*n* = 6). Scale bar, 500 μm. **h** Representative images of new bone formation assessed by dynamic histomorphometric analyses (*n* = 6). Scale bar, 25 μm. **i** Representative images of OSX (green) and OPN (red) immunostainings of OPN^+^ and OSX^+^ area on distal femurs (*n* = 6). Scale bar, 200 and 50 μm, respectively. Quantitative analysis of MAR, BFR/BS (**j**) and immunofluorescence staining of OPN and OSX (**k**) in femur tissues. **l** mRNA expressions of osteogenesis gene (*Spp1*, *Col1a1*, *Alp*, and *Bglap*) expression in tibia, tested by qPCR (*n* = 3). **m** Principal component analysis among the normal, DSS exposure, and *B. lactis* treatment group. **n** Correlation analysis of muscle function parameters and bone formation parameters among three groups. **o** Heatmap of *B. lactis* A6’s therapeutic efficacies based on indexes of muscle and bone function. Values are presented as the average ± standard deviation. The significance level (*P* value) was determined through a two-sided Welch’s *t*-test (**a**–**l**) and assessed with one-way ANOVA (**m**–**o**)

Our initial findings revealed that *B. animalis* significantly decreased following DSS exposure, prompting an investigation into its potential therapeutic relevance in bone and muscle loss. Then, our focus turned to *B. lactis* A6, a probiotic derived from an elderly individual from Bama, Guangxi, China. This particular strain, as established in our previous research, is renowned for its efficacy in enhancing digestive health, bolstering the immune system, and offering additional benefits.^30–32^ To explore the effects of *B. lactis* A6 on musculoskeletal health, we orally administered mice with or without either *B. lactis* A6 during DSS exposure (Fig. S6a). Remarkably, mice treated with *B. lactis* A6 exhibited increased body weight (Fig. S6b) and diminished serum CK levels, indicating less muscle damage (Fig. 3a). Behavioral analysis demonstrated improvements in limb strength, hanging performance, and exercise capacity (Fig. 3b). Additionally, administration of *B. lactis* A6 ameliorated muscle mass loss (Fig. 3c) and reduced the expression of muscle atrophy markers *Atrogin-1* and *Murf-1* (Fig. S6c). Histopathological analysis of via H&E staining showed less sarcolemma disruption and muscle damage with *B. lactis* A6 treatment, supported by increased CSA and minimal Feret’s diameters of muscle fibers (Fig. 3d and Fig. S6d). Immunofluorescent staining indicated increases in the number of type IIB fibers and decreases in type I fibers after *B. lactis* A6 treatment, representing a shift in muscle fiber type distribution favoring glycolytic fibers (type IIB) over oxidative fibers (type I) (Figs. 3e and S6e). This trend was notable, given that a shift toward glycolytic fibers has been previously linked with high strength, and powerful contractions.^33^

Regarding bone health, micro-CT analysis revealed that *B. lactis* A6 administration improved bone density and trabecular structure (Fig. 3f), reflected in higher BMD, BV/TV, Tb. Th and Tb. N values (Fig. 3g). Mechanical stress testing further confirmed the bone-strengthening effects of *B. lactis* A6, as indicated by increases in Young’s modulus, maximum load, and energy to ultimate load (Fig. S6f). Moreover, dynamic histomorphometric analyses (Fig. 3h) as well as immunofluorescence staining against OSX, OPN, and OCN (Fig. 3i and Fig. S6g) showed significant increases in the MAR, BFR (Fig. 3j), and the numbers of labeled osteoblasts (Fig. 3k and Fig. S6g), while TRAP staining showed a decline in the numbers of labeled osteoclasts (Fig. S6h). Followed the trend, serum bone metabolism marker showed that P1NP level increased and CTX level decreased (Fig. S6i). A qPCR analysis of femurs also showed increased levels of four pro-osteogenic genes (*Spp1*, *Col1a1*, *Alp*, and *Bglap*) after *B. lactis* A6 administration (Fig. 3l).

To provide a clearer visualization for evaluating the anti-osteosarcopenia effects of *B. lactis* A6, we further performed principal component analysis (PCA) of musculoskeletal parameters (Fig. 3m). Remarkably, the *B. lactis* A6 group exhibited a shift toward the normal group. Pearson correlation analysis revealed strong negative correlations between positive musculoskeletal parameters and the muscle damage parameter CK (Fig. 3n). Overall, the *B. lactis* A6 group showed enhanced musculoskeletal parameters and decreased muscle damage compared to the DSS group, resembling the normal group patterns (Fig. 3o). Collectively, these findings suggest that probiotic *B. lactis* A6 administration effectively alleviates bone and muscle loss.

To further investigate the role of *B. lactis A6* on bone and muscle health, we conducted an experiment using wide-spectrum antibiotics (ABX)-treated mice with or without *B. lactis* A6 supplement (Fig. S7a). The result showed that after antibiotics treatment, mice showed decreases in limb strength, hanging performance, and exercise capacity, and *B. lactis* A6 treatment reversed this process (Fig. S7b). Besides, supplement of *B. lactis* A6 ameliorated muscle mass loss after antibiotics use (Fig. S7c). Histopathological analysis of H&E staining showed larger muscle fiber with *B. lactis* A6 treatment, supported by increased minimal Feret’s diameters (Fig. S7d). Also, antibiotics treatment led to increased muscle atrophy and *B. lactis* A6 reduced the expression of muscle atrophy markers (Fig. S7e). In terms of bone health, mice treated with antibiotics showed decreased trabecular bone in the femur with reduced BMD, BV/TV, Tb. Th, along with increased BS/BV, while *B. lactis* A6 supplement reversed this trend (Fig. S7f). Additionally, by bone histomorphometric analyses, the MAR and BFR provided further evidence of impaired bone formation after antibiotics use, and a recuperative effect following *B. lactis* A6 treatment (Fig. S7g). Altogether, these results underscore the potential of *B. lactis* A6 as a multifaceted agent for combatting bone and muscle loss, offering insights into its mechanisms and therapeutic implications.

### *B. lactis* A6 ameliorates bone and muscle loss by promoting butyrate-producing bacteria and enhancing butyrate production through cross-feeding mechanism

**Fig. 4:**
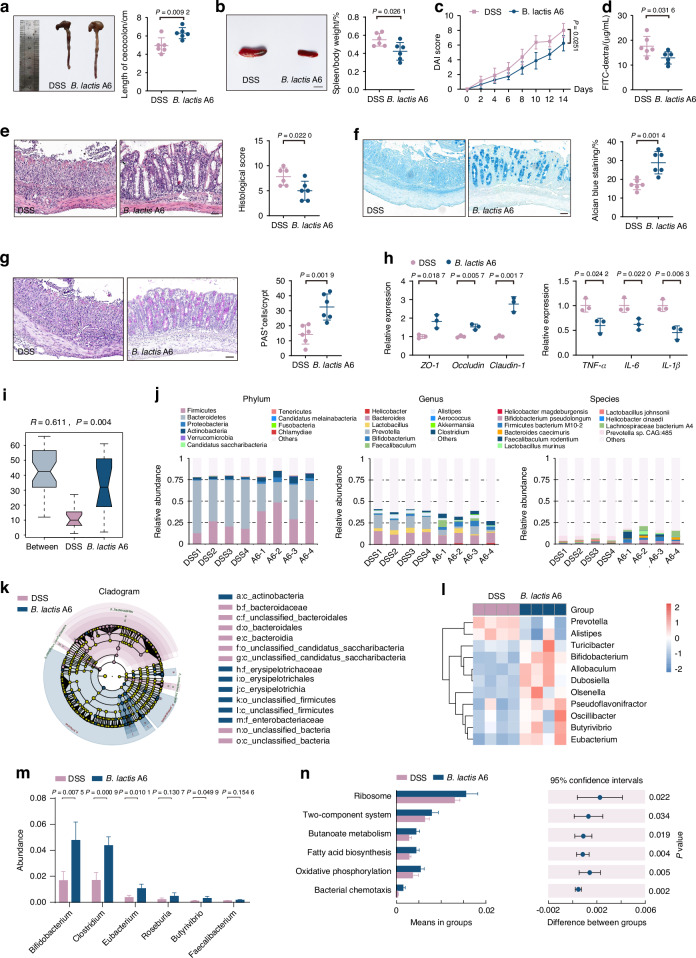
*B. lactis* A6 alleviates intestinal injury and enhances butyrate-producing bacteria composition. **a** Images and quantification of colon tissues (*n* = 6). **b** Images and quantification of spleen tissues (*n* = 6). Scale bar, 1 cm. **c** DAI score evaluation (*n* = 6). **d** FITC-dextran concentrations in serum (*n* = 6). Representative images and quantification analysis of hematoxylin and eosin (H&E) (**e**), Alcian Blue (AB) (**f**) and periodic acid Schiff (PAS) staining (**g**) from each group (*n* = 6). Scale bar, 50 μm. **h** mRNA expressions for tight junction proteins (Zonula Occcludens-1 (*ZO-1*), *Occludin*, and *Claudin-1*) (left) and inflammatory indicators (*TNF-α*, *IL-6*, and *IL-1β*) (right) in colon tissues, tested by qPCR (*n* = 3). **i** Anosim analysis based on phylum level between the two groups. **j** Structure plot of the relative fecal bacterial abundances in phylum and genus-level based on Bray–Curtis distance. **k**, **l** Analysis of cladogram generated from LEfSe and the heatmap cluster across different taxa levels. **m** Quantitative analysis of differential taxa in two groups. **n** Significant differences in metagenomic functions in *B. lactis* A6 groups compared with DSS controls based on KEGG database. Values are displayed as average ± standard deviation. Significance (*P* value) is calculated using two-way ANOVA multiple comparisons (**c**) or two-tailed Welch’s *t*-test (**a**, **b**, **d**–**h**)

Probiotics are known for favoring beneficial bacteria while suppressing pathogenic strains, thereby fostering a diverse and robust microbial ecosystem.^34,35^ Based on burgeoning evidence suggesting a gut-musculoskeletal axis,^36^ we sought to investigate whether the protective effects of *B. lactis* A6 were attributed to microbiome alterations. Following *B. lactis* A6 administration, mice exhibited elongated colorectum (Fig. 4a), reduced spleen swelling (Fig. 4b), and lower disease activity index (DAI) score (Fig. 4c). Besides, results demonstrated a significant decrease in FITC-dextran permeability, indicating less intestinal damage and enhanced gut barrier function (Fig. 4d). Pathologically, tissues from the *B. lactis* A6-treated group showed reduced intestinal wall degradation, lessened crypt injury, and decreased inflammatory cell infiltration (Fig. 4e). The mucin composition and polysaccharides distribution of colon tissues were found increased through Alcian Blue (AB) (Fig. 4f) and periodic acid Schiff (PAS) staining (Fig. 4g). These data suggest that *B. lactis* A6 strengthens the mucosal layer, improving gut barrier resilience against intestinal damage. To investigate *B. lactis* A6’s influence on the intestinal barrier, we assessed the gene expression of barrier proteins, including Zonula Occludens-1 (*ZO-1*), *Occludin*, and *Claudin-1*, and observed upregulation, signifying enhanced gut barrier integrity (Fig. 4h, left). Moreover, given the intense inflammatory response characterizing colitis, we examined the gene expressions related to inflammation in colon tissue, including tumor necrosis factor α (*TNF-α*), interleukin-6 (*IL-6*), and interleukin-1β (*IL-1β*). Our results revealed a significant downregulation of these genes following *B. lactis* A6 supplementation (Fig. 4h, right), suggesting a dampened inflammatory response in the intestinal tissue. Collectively, these findings highlight the role of *B. lactis* A6 in reducing mucosal barrier damage and subsequent inflammatory responses.

To decipher the link between *B. lactis* A6 administration and the alleviation of osteosarcopenia-like phenotypes, we conducted gut microbiota analysis following *B. lactis* A6 treatment (Fig. S8a, b). Supported by Anosim analysis (Fig. 4i), *α*-diversity values with Shannon index (Fig. S8c) and *β*-diversity with PCoA (Fig. S8d), we observed significant differences in species community between the *B. lactis* A6 and vehicle-gavage DSS-exposed mice. The composition of microbiota was profiled at various taxonomical levels. At the phylum level, *B. lactis* A6 administration led to notable increases of Firmicutes, Actinobacteria, and Proteobacteria, while decreases in Bacteroidetes (Fig. 4j, left). At the genus level, we observed increases in *Bifidobacterium*, *Faecalibaculum*, and *Clostridium*, while a decrease in *Prevotella* (Fig. 4j, middle). At species level, *B. lactis* A6 led to an increase in *B. pseudolongum*, *Firmicutes bacterium* M10-2, while a decrease was observed in *Prevotella* sp. CAG:485 (Fig. 4j, right). A cluster analysis corroborated these alterations in the microbiota composition, further substantiating the role of *B. lactis* A6 in reshaping gut microbial community (Fig. S8e). Further, LEfSe analysis revealed increased *Bifidobacterium*, *Butyrivibrio*, and *Eubacterium*, alongside decreased *Prevotella* and *Alistipes* (Fig. 4k, l and Fig. S8f).

Of note, we noticed a marked rise in the presence of advantageous bacteria that produce butyrate, including *Clostridium*, *Eubacterium*, *Roseburia*, and *Butyrivibrio* after *B. lactis* A6 treatment (Fig. 4m). Our examination of metagenomic functions using the KEGG database also showed an increased proportion related to butanoate metabolism (Fig. 4n), indicating enhanced butyrate production. Taken together, our findings propose that *B. lactis* A6 modulates gut microbiota, promotes butyrate-producing bacteria, and enhances butyrate production. These mechanisms contribute to the beneficial effects of *B. lactis* A6 in mitigating bone and muscle loss.

The production of butyrate in the gut involves a complex metabolic pathway that requires key substrates, such as acetate and lactate. Additionally, the enzyme butyryl-CoA: acetate CoA-transferase plays a crucial role in converting these substrates into butyrate^5^ (Fig. S9). Understanding the involvement of these components is essential for elucidating how *B. lactis* A6 enhances butyrate production. First, we measured the levels of butyrate in cultures of *B. lactis* A6 and *Clostridium butyricum* (*C. butyricum*) (a bacteria responsible for butyrate production, which was increased after *B. lactis* A6 treatment in our previous data) grown independently and in co-culture. Our results indicated that the butyrate levels were not significantly high when *B. lactis* A6 or *C. butyricum* were cultured independently. However, when co-cultured, the butyrate levels increased significantly (Fig. S10a, left). This finding suggests that *B. lactis* A6 facilitates the production of butyrate by *C. butyricum*. To delve deeper into the mechanistic aspects, we considered the critical factors and substrates involved in butyrate production, such as acetate and lactate. Then, we measured the levels of acetate and lactate under different conditions. When *B. lactis* A6 was cultured independently, it produced significant amounts of acetate and lactate. However, when co-cultured with *C. butyricum*, the levels of these substrates decreased, suggesting their consumption for butyrate production (Fig. S10a, middle and right). This indicates that *B. lactis* A6 facilitates butyrate production by supplying necessary substrates to *C. butyricum*. To further confirm the role of these substrates and to eliminate potential confounding factors arising from bacterial interactions, we conducted supplementation experiments. The acids in the supernatant from *B. lactis* A6 cultures were neutralized and then tested for its effect on butyrate production by *C. butyricum* (Fig. S10b). Our results showed that the unneutralized supernatant from *B. lactis* A6 promoted butyrate production by *C. butyricum*. However, once the supernatant was neutralized, its ability to promote butyrate production was lost. When the neutralized supernatant was supplemented with acetate, lactate, or both, the ability to promote butyrate production was partially restored, with acetate showing a more significant effect than lactate (Fig. S10c). This indicates that acetate and lactate play critical roles in the butyrate production process by *C. butyricum*, with acetate being more important. Besides, in vivo relevance of these findings was confirmed by administering *B. lactis* A6 to mice and analyzing the luminal contents of the ileum (Fig. S10d). Measurement of the transcript levels of butyryl-CoA: acetate CoA-transferase revealed significant upregulation in the *B. lactis* A6-administered group compared to the control group (Fig. S10e). This suggests that *B. lactis* A6 promotes butyrate production in the gut microbiota through a cross-feeding mechanism with *C. butyricum* and the modulation of butyrate metabolic pathways.

### *B. lactis* A6 reverses osteosarcopenia-linked butyrate depletion in serum, muscle, and bone tissues

**Fig. 5:**
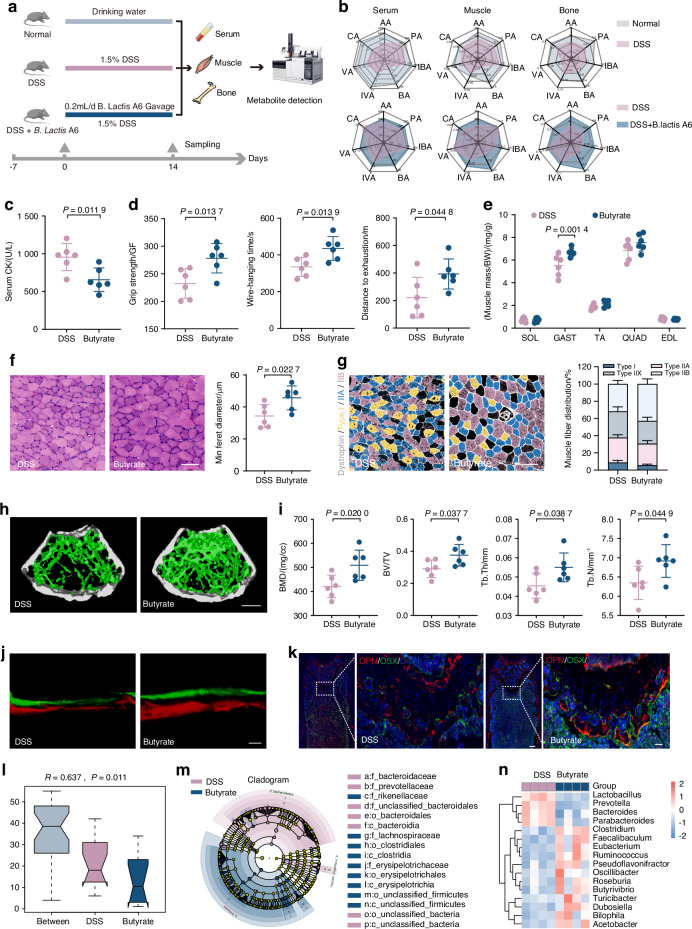
*B. lactis* A6-favored butyrate alleviates bone and muscle and enriches beneficial SCFA-producing bacteria. **a** Schematic representation illustrating the experimental design. Short-chain fatty acids (SCFAs) were detected in serum, muscle, and bone through GC–MS analysis. **b** Mean compositions of SCFAs in serum, muscle, and bone from the normal and DSS group (upper), and from the DSS and *B. lactis* A6 group (down) (*n* = 6). **c** Measurement of CK activity of the serum (*n* = 6). **d** Assessment of physical performance by handgrip, hanging wire tests, and treadmill, respectively (*n* = 6). **e** Muscle mass analysis from each group (*n* = 6). **f** H&E staining images in gastrocnemius cross-sections and quantification of average minimal Feret’s diameter of myofibers (*n* = 6). Scale bar, 100 μm. **g** Representative immunofluorescence images to visualize specific types of muscle fibers and fiber type compositions (*n* = 6). Scale bar, 100 μm. **h** Representative micro-CT images of metaphyseal bone. **i** Quantitative analysis of bone mass (*n* = 6). Scale bar, 500 μm. **j** Representative images and quantification of new bone formation assessed by dynamic histomorphometric analyses (*n* = 6). Scale bar, 25 μm. **k** Representative images of OSX (green) and OPN (red) immunostainings of OPN^+^ and OSX^+^ area on distal femurs (*n* = 6). Scale bar, 200 and 50 μm, respectively. **l** Anosim analysis based on phylum level between the DSS and butyrate group. **m**, **n** Analysis of cladogram generated from LEfSe and the heatmap cluster across different taxa levels. Values are represented as the average ± standard deviation. The significance level (*P* value) was determined through a two-sided Welch’s *t*-test

Given increased butyrate-producing bacteria and heightened butanoate metabolism, we hypothesized butyrate’s pivotal function in mediating musculoskeletal development. To investigate this, we focused on gut metabolites, particularly short-chain fatty acids (SCFAs).^37,38^ We employed GC–MS analysis to assess SCFA levels in serum, muscle (Gast), and bone (femur) samples obtained concurrently from normal, DSS-exposed, and *B. lactis* A6-treated mice (Fig. 5a). DSS-exposed mice exhibited significant reductions in acetic acid, caproic acid, isovaleric acid, and notably, butyrate levels in serum. Similar trends were observed in muscle, with DSS-exposed mice showing significant decreases in propionic acid, valeric acid, and butyrate. In bone, substantial reductions were noted in butyrate, valeric acid, and caproic acid levels (Fig. 5b, upper and Table S4). Importantly, butyrate levels uniformly decreased across all examined tissues in serum, muscle, and bone. This consistent depletion suggests that changes in gut metabolite profiles resulting from DSS exposure underpin the emergence of bone and muscle loss. Notably, following *B. lactis* A6 treatment, butyrate levels across serum, muscle, and bone tissues experienced a significant recovery (Fig. 5b, down and Table S5). This observation aligns with our microbiota analysis and supports our hypothesis that alterations in butyrate levels closely correlate with bone and muscle loss, underscoring the therapeutic importance of *B. lactis* A6 in reversing this change.

### Butyrate supplementation alleviates bone and muscle loss and protects intestinal barrier function

With a consistent butyrate decrease in serum, muscle, and bone after DSS exposure, and considering the critical role of butyrate in diseases including rheumatoid arthritis,^39^ acute myeloid leukemia,^40^ etc., we further explored its therapeutic potential for bone and muscle loss. After administrating butyrate to mice (Fig. S11a), we observed notable gains in body weight (Fig. S11b) and reductions in serum CK levels (Fig. 5c). Behavioral analyses revealed improvements in muscle functions (Fig. 5d), and increases in Gast mass (Fig. 5e). qPCR analysis also showed reduced expression of atrophy markers (Fig. S11c). Besides, H&E staining confirmed improved muscle integrity and increased muscle fiber size after butyrate supplementation (Fig. 5f and Fig. S11d). Immunofluorescence staining outcomes further underscored a significant rise in type IIB fibers and a reciprocal decrease in type I/type IIA fibers (Fig. 5g and Fig. S11e). For bone, micro-CT data showed enhanced skeletal density and structure in butyrate-treated mice, with higher BMD, BV/TV, Tb. Th, and Tb. N measurements (Fig. 5h, i). Mechanical stress testing indicated enhanced bone strength (Fig. S11f). Dynamic histomorphometric analyses (Fig. 5j) and immunofluorescence staining (Fig. 5k) showed significant increases in the MAR, BFR (Fig. S11g), and the numbers of OSX-, OCN- and OPN-positive osteoblasts (Fig. S11h, j) in the butyrate-gavaged mice, while the numbers of TRAP-positive osteoclasts decreased (Fig. S11k). Serum bone metabolism analysis revealed elevated serum levels of PINP, signifying heightened bone formation, whereas diminished levels of CTX indicate decreased bone resorption (Fig. S11l). qPCR analysis also demonstrated upregulated pro-osteogenic genes (Fig. S11i). These findings support the beneficial role of *B. lactis* A6-favored butyrate in mitigating gut dysfunction-induced bone and muscle loss.

The intestinal tract is the main location for butyrate synthesis, where it chiefly serves as fuel for epithelial cells, enhancing intestinal barrier function.^41^ To investigate whether butyrate could preserve gut barrier function, we administered butyrate to DSS-exposed mice. Results showed that butyrate administration led to elongated colorectum (Fig. S12a), diminished spleen swelling (Fig. S12b), and decreased DAI score (Fig. S12c). We also found that these mice exhibited improved gut barrier integrity, as indicated by reduced FITC-dextran permeability (Fig. S12d). Further, colon pathological evaluation presented lesser crypt damage (Fig. S12e), more expansive mucin (Fig. S12f), and wider distribution of polysaccharides in the intestinal mucosa (Fig. S12g). Aligned with these findings, tight junction molecules were observed upregulated (Fig. S12h), and inflammatory indicators downregulated (Fig. S12i). In summary, these results indicated that butyrate protects gut barrier function, reduces endothelial damage, and consequently mitigates bone and muscle loss.

Studies have revealed microbiome alterations in volunteers consuming butyrylated high amylose maize starch, implying that butyrate selectively modulates gut bacteria.^42,43^ Through metagenomics sequencing (Fig. S13a, b), we observed noteworthy differences in species abundance, validated by Anosim analysis (Fig. 5l), *α*- and *β*-diversity values (Shannon and Simpson index) (Fig. S13c, d) between butyrate and vehicle-gavage group. Following butyrate treatment, the LEfSe analysis indicated augmented levels in genera including *Clostridium*, *Faecalibaculum*, *Eubacterium*, *Roseburia*, and *Butyrivibrio*, while noting diminished levels in *Lactobacillus*, *Prevotella*, and *Bacteroides* (Fig. 5m, n and Fig. S13e). Microbiome alterations were further profiled at multiple taxonomic levels through cluster analysis (Fig. S13f, g). Many of the genera that increased, such as *Clostridium*, *Eubacterium*, *Roseburia*, *Butyrivibrio*, and *Faecalibaculum*, contain species known to produce butyrate or other SCFAs that are beneficial to gut health (Fig. 5n). These alterations may enhance SCFA concentrations, potentially benefiting gut barrier function and reducing inflammation. Overall, they support the protective effects of *B. lactis* A6-favored butyrate supplementation against intestinal injury and dysfunction, suggesting synergy between butyrate, gut microbes, and digestive health.

### Butyrate reduces inflammation in T cells and inhibits NF-κB pathway activation

**Fig. 6:**
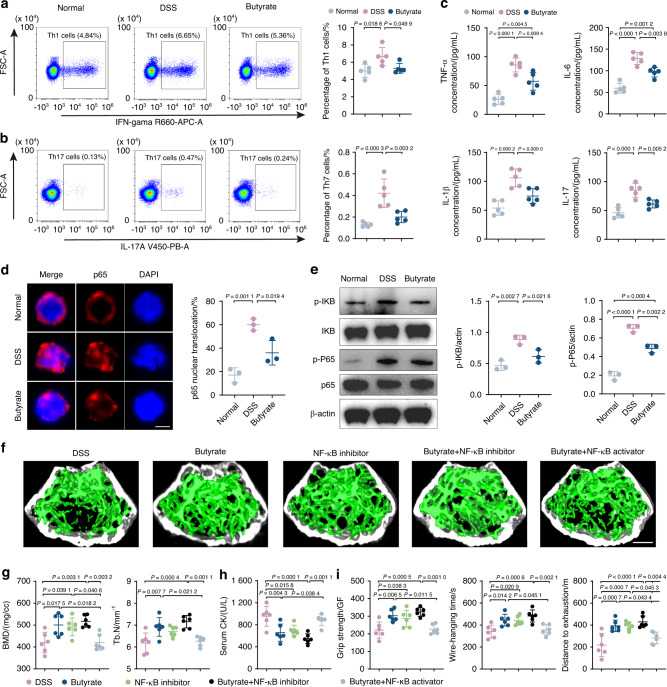
Butyrate reduces inflammation in T cells and inhibits NF-κB pathway activation. **a, b** Percentages of Th1 and Th17 cells detected by flow cytometry (*n* = 5). **c** Concentration of TNF-a, IL-6, IL-1β, and IL-17 in serum analyzed by ELISA (*n* = 5). **d** Immunofluorescence with NF-κB antibody in T cells from normal, DSS-exposed, and butyrate-treated mice. Immunopositive cells for nuclear NF-κB were quantified as percent of total cells (*n* = 3). Scale bar, 5 μm. **e** Representative WB images and quantitative analyses of p-P65, P65, p-IκB, and IκB (*n* = 3). Micro-CT images (**f**) and quantification of BMD, Tb. N (**g**) in DSS group, butyrate-treated group, NF-κB inhibitor group, butyrate + NF-κB inhibitor group and butyrate + NF-κB inhibitor group. Scale bar, 500 μm. Quantification of serum CK (**h**), grip strength, wire-hanging time, and distance to exhaustion (**i**) in different groups. Values are represented as the average ± standard deviation. The significance level (*P* value) was assessed with one-way ANOVA

In our previous investigation,^44^ we observed a common transition toward pro-inflammatory phenotypes in CD4^+^ T cells across tissues during the process of inflammaging. These cell populations were characterized by an enrichment of genes associated with pro-inflammatory IL-17 and TNF signaling pathways, along with evident pro-inflammatory traits and heightened cytotoxicity. Notably, the nuclear factor kappa B subunit (NF-κB), a core component of the NF-κB signaling pathway, emerged as a direct downstream target in both Th1-like and Th17 cells, implicating its pivotal role in orchestrating the pro-inflammatory transition in T cells. In this study, we observed a synergistic recovery effect of butyrate intervention on muscles and bones, leading us to hypothesize the presence of an upstream common regulatory mechanism. Specifically, we sought to elucidate whether butyrate influences the shared pro-inflammatory state in T cells and whether this modulation is mediated by NF-κB activation. In our previous study,^44^ CD4^+^ T cells in aged muscle experience Th1-like differentiation, and, in bone, a skewing toward Th17 cells was observed. Then, to determine whether Th1 and Th17 cells also exhibit a change after butyrate treatment, we isolated T cells from bone marrow and detected them through flow cytometry. In our study, we found that the proportion of Th 1 and Th17 cells, which are responsible for pro-inflammatory features, were higher in the DSS-exposed group than the normal group and this trend was reversed with the butyrate supplementation (Fig. 6a, b). This indicates that DSS-exposure turn T cells into pro-inflammatory phenotype in the bone marrow, which may play a potential role in the change of bone and muscle, validating the role of T cells in mediating inflammaging, as elucidated in our previous study.^44^ Furthermore, butyrate administration attenuated inflammation-induced bone and muscle loss by regulating the activity of Th1 and Th17 cells. Of note, various factors including reactive oxygen species and cytokines such as TNF-α, IL-6, and IL-1β can activate the inflammatory response. Consistent with our hypothesis, we observed a decline in the serum concentration of inflammatory cytokines, including TNF-α, IL-6, IL-1β, and IL-17, following butyrate treatment (Fig. 6c). This alteration revealed a shift in bone marrow T cells toward a pro-inflammatory phenotype in the context of DSS-induced gut dysfunction. This shift was significantly mitigated by butyrate supplementation, indicating a direct influence of butyrate on modulating immune cell behavior, thereby potentially contributing to the observed synergistic effect on muscle and bone health.

The NF-κB is a pleiotropic transcription factor that has been reported to be closely associated with inflammation-related dysfunction in various contexts.^45^ In our previous investigation,^44^ the NF-κB signaling pathway was found as a direct downstream target in both Th1-like and Th17 cells and orchestrated the pro-inflammatory transition in T cells. Since NF-κB is pivotal in regulating T cell phenotype, particularly through phosphorylation of p65, a critical step in the NF-κB signaling cascade leading to nuclear translocation and subsequent transcriptional activation, we isolated T cells from bone marrow to assess the inflammatory NF-κB levels. Although the relationship between gut microbiota-derived butyrate and NF-κB is complex, it is generally accepted that butyrate suppressed NF-κB activation and reduce NF-κB-mediated inflammatory signaling. In contrast, multiple investigations have demonstrated that gut dysfunction can support NF-κB activation. The possible reason for this is that gut dysfunction increased the phosphorylation of IκB, triggering its degradation and allowing NF-κB to enter the nucleus. According to our research, DSS-exposed T cells exhibited an increased proportion of p65 protein entry into the nucleus (Fig. 6d), as well as increased protein expression of p-IκB and p-P65 compared to the control group (Fig. 6e). This indicates that NF-κB pathway was turned on by DSS exposure. The addition of butyrate, however, resulted in decrease in the proportion of NF-κB entering the nucleus, as well as p-IκB and p-P65 protein expression (Fig. 6d, e). The data above indicated that butyrate can counteract the activation of NF-κB in T cells caused by DSS-induced gut dysfunction. This finding supports the notion that butyrate alleviated inflammation-induced bone and muscle loss through reducing T cell pro-inflammatory activity and inhibiting NF-κB signaling activation.

To further figure out whether these changes in the NF-κB pathway mediated the butyrate-induced improvements in bone and muscle phenotypes, we also established five experimental groups: DSS control, butyrate-treated, NF-κB inhibitor, butyrate combined with NF-κB inhibitor, and butyrate combined with NF-κB activator group. After treatment, micro-CT analysis of trabecular bone showed a marked improvement in bone density and trabecular structure in the butyrate-treated group compared to the DSS control (Fig. 6f). The NF-κB inhibitor group and the group treated with butyrate and the NF-κB inhibitor also displayed significant bone loss alleviation. Quantification of BMD and Tb. N further supported these findings (Fig. 6g). Serum CK levels and behavioral analyses in limb strength, hanging performance, and exercise capacity were measured to assess muscle health. The butyrate-treated group showed lower serum CK levels and improved muscle functions compared to the DSS group. The NF-κB inhibitor group and the group treated with butyrate and the NF-κB inhibitor showed great muscle health indicators. (Fig. 6h, i). Interestingly, while both butyrate and the NF-κB inhibitor alleviated bone and muscle loss individually, their combination did not produce a synergistic effect, suggesting that NF-κB is a critical target in the butyrate pathway. This was further confirmed by the observation that co-treatment with butyrate and the NF-κB activator negated the protective effects of butyrate, reinforcing the conclusion that butyrate’s therapeutic effects are mediated through NF-κB pathway modulation (Fig. 6f–i). These results indicate that butyrate effectively reduces bone and muscle loss through modulation of the NF-κB pathway. Targeting the NF-κB pathway could be a crucial strategy in enhancing butyrate’s therapeutic efficacy in musculoskeletal health.

## Discussion

Muscle and bone constitute significant portions of body mass, crucial for movement and metabolic health.^46,47^ Global biological processes, including metabolism, inflammation, and aging, strongly impact the stability of these tissues. Traditional musculoskeletal research primarily focuses on isolated bone or muscle pathologies. In our study, we prioritize investigating the impact of gut dysfunction on both skeletal muscle and bone health, bridging mechanobiology with broader physiological responses. Despite prior studies observing on isolated bone or muscle pathologies with IBD,^15,16^ the mechanisms through which gut alterations drive these losses and potential therapeutic approaches remain unclear. Our research contributes by revealing distinct microbiota imbalances associated with bone and muscle loss and their active role in its development. This deepens our understanding of the gut’s interplay with the bone and muscle systems, offering insights for specialized therapeutic strategies. We employed the DSS-induced colitis model, mimicking human IBD symptoms,^48^ to explore how compromised gut function affects bone and muscle health. Delving into the relationship between the gut and bone and muscle, we identified shifts in microbial compositions, particularly a reduction in *Bifidobacterium* taxa, closely linked to bone and muscle deterioration.

The gut-bone and muscle axis theory, a well-established concept, posits that intestinal microbiota influence nutrient metabolism and the host’s immune response, subsequently impacting bone and muscle health.^49,50^ For instance, earlier research has emphasized the crucial role played by the gut microbiota in determining bone and muscle structure and functionality.^11,12^ Kim et al. identified that introducing gut microbiota from younger mice rejuvenated the physiological capabilities of older mice, which manifested in changed gut microbial dynamics and altered genetic activity within muscles and skin.^51^ Probiotic-based interventions hold promise as a therapeutic approach for bone and muscle loss. Building upon the observation of *B. animalis* decline following DSS exposure, we investigated whether *B. lactis* A6 could be utilized as a potential therapeutic intervention for bone and muscle loss. Our findings demonstrated that *B. lactis* A6 significantly ameliorated bone and muscle loss, suggesting its clinical relevance and highlighting implications for personalized medicine in the field of musculoskeletal diseases. Additionally, a closer look into *B. lactis* A6’s therapeutic dynamics revealed its connection to butyrate. Butyrate was also reported to be associated with musculoskeletal health. Tyagi et al. showed that oral supplementation with probiotic *Lactobacillus rhamnosus* GG (LGG) increases bone mass in mice by increasing butyrate. LGG or butyrate increases the frequency of regulatory T (Treg) cells and stimulates bone formation by activating Wnt signaling in osteoblasts.^52^ Also, Tang et al. found that butyrate exerts protective effects on muscle atrophy induced by diabetic nephropathy by enhancing intestinal barrier function and activating the FFA2 receptor-mediated PI3K/Akt/mTOR pathway.^53^ Even though *B. lactis* A6 does not produce butyrate on its own, post *B. lactis* A6 administration, we recorded elevated levels of bacteria known for butyrate production, including members like *Clostridium*, *Eubacterium*, *Roseburia*, and *Butyrivibrio*.^54^ Furthermore, our metagenomic functional insights detected a rise in the metabolic activities related to butanoate, suggesting a probable augmentation in butyrate generation. Research indicates that the majority of SCFAs, butyrate included, originate from the metabolic processes of gut bacteria.^52,55^ In our study, we found that butyrate concentrations were significantly decreased after DSS exposure, with subsequent *B. lactis* A6 administration restoring butyrate levels in serum, muscle, and bone, underscoring its pivotal role in *B. lactis* A6’s therapeutic efficacy against bone and muscle loss. Validating this, we also employed butyrate supplementation and affirmed its potential in counteracting bone and muscle loss. Our findings indicate that *B. lactis* A6’s anabolic effect is due to its synergy with the existing microbiome in butyrate production. Further research is required to elucidate *B. lactis* A6’s influence on other SCFAs and their contributions to its role in enhancing bone and muscle health.

IBD is a debilitating disease that imposes a burden on the body, resulting in a caloric deficit, less protein synthesis, and more protein breakdown, leading to muscle atrophy. While our study establishes a correlation between altered gut microbiota and muscle atrophy, supported by data showing microbial imbalances and butyrate production deficits in DSS-exposed mice, we acknowledge that IBD-related muscle wasting is likely multifactorial. This condition is not singular in its causation but rather involves a complex interplay of factors. Persistent inflammation, characteristic of IBD, likely affects systemic processes, leading to the upregulation of catabolic signaling and an increase in pro-inflammatory cytokines like TNF-a and IL-6, which are known to promote musculoskeletal protein breakdown.^56^ Concurrently, IBD’s impact on intestinal functionality can severely impair nutrient absorption, leading to deficiencies in essential amino acids and other nutrients critical for bone and muscle repair and growth.^57^ The disease can also disturb the balance of anabolic and catabolic hormones, with decreased levels of hormones such as IGF-1 and increased levels of cortisol, further contributing to muscle atrophy.^58^ Additionally, the pain and fatigue associated with IBD symptoms, and cellular damage to bone and muscle due to elevated oxidative stress can significantly reduce physical activity levels, exacerbating bone and muscle loss through disuse atrophy mechanisms.^59,60^ These aspects collectively underscore the complexity of IBD-related muscle wasting, suggesting that it may also be a condition influenced by a diverse array of biological and physiological factors.

Maintaining gut barrier integrity holds significant therapeutic potential in addressing bone and muscle loss. The intestinal epithelial barrier is essential for preventing the translocation of harmful substances and bacteria from the gut lumen to the bloodstream.^61^ Disruption of this barrier, as observed in IBD, can lead to heightened gut permeability, chronic inflammation, and tissue damage.^62^ In our study, we demonstrated that *B. lactis* A6-favored butyrate significantly improved intestinal integrity and mitigated intestinal damage. This aligns with butyrate’s role in supporting the gastrointestinal epithelium and enhancing the stability of the intestinal lining.^55^ Moreover, pathological evaluations and genetic expression analyses suggest that the butyrate supported by *B. lactis* A6 plays a part in upholding gastrointestinal lining function, facilitating the expression of tight junction proteins, and curbing widespread inflammation. By analyzing the microbiota following butyrate supplementation, we found that species known to produce butyrate or other SCFAs that are beneficial to gut health were increased, which further augmented the concentration of butyrate in the gut, potentially enhancing gut barrier function and reducing inflammation. Specifically, this healthier gut environment could mean less oxidative stress, which could potentially benefit the anaerobic butyrate producers, and suggests a positive feedback loop between butyrate, gut microbiota, and gut health.

In our study, we specifically focused on the role of *B. lactis* A6 in modulating gut dysfunction and its consequent muscle and bone loss. The selection of *B. lactis* A6 as our research focus was not arbitrary but based on its significant benefits demonstrated in our previous investigations. In our previous studies, we have isolated *B. lactis* A6 from an elderly individual from Bama, Guangxi, China, and demonstrated that this probiotic showed effectiveness in restoring intestinal barrier function, modulating immune responses, and enhancing gut health. In recent years, research on *B. lactis* A6 has substantially increased, revealing its potential roles in the prevention and treatment of various diseases, particularly those related to gut health. For instance, our previous study has found that *B. lactis* A6 can improve intestinal barrier function, reduce oxidative stress in colon tissue and suppress inflammatory responses in the colon in DSS-induced mice with IBD.^32^ Additionally, by enhancing intestinal butyric acid concentrations and triggering the butyric acid-FFAR pathway, it mitigated colitis caused by DSS, thereby playing a crucial role in maintaining intestinal health.^63^ Notably, researchers discovered that the efficacy of *B. lactis* A6 extends beyond intestinal health to encompass general health. *B. lactis* A6 alleviates obesity associated with promoting mitochondrial biogenesis, fatty acid β-oxidation of adipose tissues in mice with diet-induced obesity.^64,65^ Together, our study’s choice to focus on *B. lactis* A6 is based on its demonstrated broad benefits in previous research, including improving gut function, its potential therapeutic effects on IBDs, and its potential impacts on overall health. By delving into the role of *B. lactis* A6 in modulating gut microbiota and enhancing butyrate production, our research further substantiates the significant potential of this strain in preventing and treating musculoskeletal diseases, paving the way for future translational and clinical research endeavors.

Although butyrate supplementation provided relief for bone and muscle loss in our study, the clinical application of butyrate has certain concerns when compared to probiotic *Bifidobacterium* administration. First, butyrate supplementation has not been fully established as safe and effective in clinical practice, with concerns raised about potential side effects, such as gastrointestinal discomfort and diarrhea.^55^ Additionally, butyrate has a foul and lasting odor and taste, and its quick metabolism by the gut epithelium limits its systemic effects and requires frequent dosing.^55,66,67^ In human trials, intrarectal delivery of butyrate has shown moderate efficacy in treating diseases like colitis but is not the preferred delivery route.^68^ In contrast, probiotics have the potential to colonize the gut and interact with the host for long-term benefits. They can adjust the structure of the intestinal microbial community with greater efficacy, introducing favorable bacteria and outcompeting detrimental ones, resulting in a richer and more consistent microbiota. Moreover, probiotics can produce metabolite (e.g., butyrate) as byproducts of their fermentation, which may result in sustained butyrate production and improved gut and musculoskeletal health. Although *B. lactis* A6 alone, does not produce butyrate, *B. lactis* A6 expands intestinal butyrate-producing bacteria, indicating that *B. lactis* A6 indirectly increases SCFAs production by the gut microbiota.^34,35^ Besides, some other butyrate-producing bacteria such as *Clostridium* or *Butyrivibrio* have been used for the treatment or prevention of digestive system-related diseases such as diarrhea, intestinal infections, intestinal inflammation, and constipation.^69^ In our research, the selection of *B. lactis* A6 was strategic for several reasons. *B. lactis* A6 boasts a favorable safety profile, crucial for probiotic applications. Unlike certain strains of *Clostridium* species, which may harbor genes encoding toxins like botulinum neurotoxin complex or toxins plasmids,^70^ *B. lactis* A6 exhibits a commendable safety record, ensuring minimal risk of virulence factors transmission. Moreover, *B. lactis* A6 demonstrates robust efficiency and stability in various environments. Its ability to survive and thrive in the gut, coupled with its capacity to modulate gut microbiota and promote butyrate production, underscores its efficacy as a probiotic.^32,64,65^ Additionally, selected strains of *Bifidobacterium* species, such as *B. lactis* A6, are commonly used probiotics and can be added to food supplements and foods (especially dairy products). In contrast, for butyrate-producing bacteria, it should be noted that there is still safety concern about the exotoxin secretion of some *Clostridium* species, like alpha-toxin and enterotoxin from *Clostridium* *perfringens*, as well as toxin A and toxin B from *Clostridium* *difficile*.^71,72^ Strict safety assessments are imperative to mitigate risks associated with virulence factors transmission and antibiotic resistance gene transfer. Furthermore, enhancing the efficiency of *Clostridium* species as probiotics necessitates addressing challenges related to adhesion, stress resistance, and strain-specificity.^70^ Strategies such as selecting strains with high adhesion ability and spore-forming capability, as well as exploring combined utilization with other probiotics or prebiotics, are essential to maximize their therapeutic potential. Recognizing individual differences in dietary habits, age, physiological state, and microbial composition is vital for optimizing the applicability of *Clostridium* species.^71–73^ In conclusion, while butyrate-producing bacteria such as *Clostridium* species exhibit promising potential as probiotics, their application entails overcoming safety concerns and optimizing efficiency. In contrast, *B. lactis* A6 emerges as a compelling probiotic candidate, offering a favorable safety profile, efficient therapeutic action, and targeted efficacy in mitigating bone and muscle loss associated with gut dysfunction. Further research and clinical trials are warranted to elucidate the comparative effectiveness and applicability of these probiotic strains in diverse therapeutic contexts.

Taken together, our research advances understanding of the gut-bone/muscle interplay, highlighting the therapeutic potential of *B. lactis* A6 in mitigating bone and muscle loss by modulating gut microbiota and enhancing butyrate production. These findings provide a foundation for future translational studies to develop microbiome-based approaches for musculoskeletal disease management, necessitating further investigations into underlying mechanisms and clinical trials to fully ascertain their therapeutic efficacy.

## Materials and methods

### Ethics statement

The experiments involving animals or humans were approved by the Ethics Committee of Chinese PLA General Hospital (Approval No. 2022-X18-11). Patients and/or the public were not involved in the design, conduct, reporting, or dissemination plans of this research. All patients and healthy volunteers signed a consent form approved by the local institutional review board.

### Animals care and study approval

Male C57BL/6J mice aged 10 weeks were acquired from Vital River Laboratories, Beijing, China, and were given a 7-day acclimation period prior to any experimental procedure. Mice were kept in an environment maintaining a temperature of 25 ± 2 °C, 50 ± 5% humidity, and a 12-h light/dark cycle. For the DSS exposure experiment, after 1 week of acclimation, mice were randomly divided into the normal and DSS groups. The normal group was treated with autoclaved water, whereas the DSS group was treated with 1.5% (w/v) DSS (36–50 kD; MP Biomedicals). On the 14th day, the mice were sacrificed by cervical dislocation. For the *B. lactis* A6 and butyrate treatment experiment, the *B. lactis* A6 group was treated with DSS and daily gavaged with 0.2 mL of 2.0 × 10^10^ CFU/mL *B. lactis* A6, and the butyrate group was treated with DSS and daily gavaged with 0.2 mL of 30 mg/kg butyrate. For the FMT experiment, the component involved the use of DSS-induced mice as subjects to assess the effects of different microbiota sources on bone and muscle health. Specifically, the experimental design included four intervention groups: (1) DSS-FMT: fecal material from DSS-induced mice (male C57BL/6J mice aged 10 weeks who suffered from DSS exposure) was transplanted back into other DSS-induced mice to serve as a control group, aimed at evaluating the impact of a consistent microbial environment under DSS-induced conditions; (2) Normal-FMT: fecal material from healthy mice (male C57BL/6J mice aged 10 weeks) was transplanted into DSS-induced mice to assess the therapeutic potential of “normal” microbiota; (3) Young-FMT: young mice’s (male C57BL/6J mice aged 10 weeks) fecal material was used for transplantation into DSS-induced mice to explore the effects of young microbiota on compromised physiological models; (4) Aged-FMT: aged mice’s (male C57BL/6J mice aged 24 months) fecal material was transplanted to study the influence of aged microbiota on skeletal health in the context of DSS-induced stress. For the wide-spectrum antibiotics-treatment experiment, 10-week-old male C57BL/6J mice were randomized into three distinct groups. (1) PBS group: mice received oral administration of PBS for 2 weeks, followed by a continuous 4-week PBS treatment. (2) ABX-PBS group: mice were administered a broad-spectrum antibiotic solution (ABX) orally for 2 weeks to disrupt the gut microbiome, followed by 4 weeks of PBS to monitor the effects of microbiome depletion on bone and muscle without microbial intervention. (3) ABX-*B. lactis* A6 group: mice underwent the same initial 2-week antibiotic treatment to alter the gut flora, subsequently receiving oral *B. lactis* A6 probiotics for 4 weeks to assess the probiotic’s therapeutic efficacy in reversing bone and muscle deterioration caused by microbiome imbalance. For broad-spectrum ABX treatment, ampicillin (1 g/L), vancomycin (5 mg/mL), neomycin (10 mg/mL), metronidazole (10 mg/mL), and amphotericin-B (0.1 mg/mL) were dissolved in the drinking water as previously described.^74,75^ On the 6th week, the functional performance of mice was evaluated and the tissues were harvested for further analysis. To explore the role of NF-κB in the butyrate-mediated alleviation of bone and muscle loss, mice were divided into five groups: DSS control, butyrate-treated, NF-κB inhibitor, butyrate combined with NF-κB inhibitor, and butyrate combined with NF-κB activator groups. The butyrate-treated group received daily gavage with 0.2 mL of 30 mg/kg butyrate. The NF-κB inhibitor group received an NF-κB inhibitor (Bay-117082, CalBiochem, injected at 10 mg/kg body weight intraperitoneally as indicated^76^) and the butyrate combined with NF-κB inhibitor group received both treatments concurrently. The butyrate combined with NF-κB activator group received an NF-κB activator (lipopolysaccharide, Sigma-Aldrich, injected at 250 μg/kg body weight intraperitoneally as indicated^77^) along with butyrate treatment.

### Human microbiota collection

Subjects in all cohorts fulfilled the following inclusion criteria^78^: (1) male or female, over the age of 18, provided informed consent; (2) not following any interventional diet modification. Furthermore, inclusion criteria for IBD patients also included clinical diagnosis of IBD based on standard of care investigations, consistent with international practice guidelines, confirmed and documented by an investigator. Exclusion criteria for IBD patients were: (1) antibiotic treatment within 3 months of consent; (2) patients with a history of colorectal cancer or cholangiocarcinoma within 1 year of consent; (3) recent clinically documented infection or active clinically significant systemic inflammatory condition unrelated to IBD; (4) pregnant or lactating women. Exclusion criteria for healthy individuals were: (1) antibiotic or probiotic use in the previous 3 months; (2) self-reported acute or chronic infectious disease; (3) background of gastrointestinal disease, under active medical treatment or follow-up, including IBD (CD, UC), celiac disease, intestinal surgery (other than historic appendectomy and gallbladder removal); (4) background of significant limiting autoimmune disease, kidney disease, metabolic disorder, endocrine disorder, cardiovascular disease, lung disease, neurological disease; (5) chronic medication usage. Following inclusion/exclusion criteria, patients did not undergo antibiotic treatment at least starting from 3 months prior the beginning of the study.

### Bacterial culture

*B. lactis* A6 (CGMCC, No. 9273) and *C. butyricum* (ATCC, 25755) were cultured both independently and in co-culture to investigate the cross-feeding mechanism and its impact on butyrate production. *B. lactis* A6 was grown in de Man, Rogosa and Sharpe (MRS) broth, while *C. butyricum* was cultured in Reinforced Clostridial Medium (RCM). For co-culture experiments, both bacterial strains were inoculated together in MRS-RCM mixed medium and incubated anaerobically at 37 °C.

### Assessment of gut dysfunction

Throughout the DSS treatment period, we consistently documented the body weight (BW) of every mouse. We then determined the percentage shift in BW in comparison to their weight before the initiation of DSS treatment. Using metrics like body weight reduction (0–4), consistency of stool (0–3), and the presence of blood in feces (0–4),^79^ as detailed in Table S1, we calculated the DAI. After sacrificing, each mouse’s colon was rinsed with chilled PBS, after which we assessed its length. For subsequent RNA extraction, sections from the central parts of the colons were preserved in RNAlater (Sigma-Aldrich, USA) and kept at −20 °C. The distal colons underwent fixation using Carnoy’s fluid followed by histological staining procedures. In addition, we documented the spleen’s weight for every mouse and then calculated the spleen-to-BW ratio.

### Histological analysis of colon tissues

The distal colon samples, after fixation in Carnoy’s solution for a duration of 16 h, were processed in paraffin. These samples were subsequently sectioned at 5 μm thickness and stained with H&E. For each sample, we evaluated the histological scores from six fields chosen at random, grading them on parameters such as the intensity of inflammation (0–3), extent of the inflammatory response (0–3), and crypt impairment (0–4). Table S2 offers a comprehensive breakdown of the histological scoring guidelines, which have an overall range between 0 and 10. Additionally, the colonic mucin distribution was highlighted using Alcian Blue staining, while goblet cells were visualized using the PAS staining method, following previously established protocols.^80^

### qPCR

We extracted total RNA employing the RNA isolator Total RNA Extraction Reagent (Vazyme, R401). For reverse transcription, we utilized 1 μg of the total RNA, combined with the HiScript III RT SuperMix designated for qPCR (Vazyme, R323). We prepared amplification reactions in a 20 μL mixture, which included 1 μL of cDNA, the ChamQ Universal SYBR qPCR Master Mix (Vazyme, Q711), and the relevant primers. Each reaction was carried out in triplicate and the results were then averaged. For a detailed list of the primers we used in this research, refer to Table S3. We gauged the relative abundance of the target gene by contrasting it with the β-actin gene, making use of the 2^−ΔΔCT^ technique.^81^

### Metagenomic analysis

For the preprocessing of raw Illumina sequencing data and deriving clean data for further investigation, we employed Readfq (V8, https://github.com/cjfields/readfq). We analyzed the metagenome assembly of this clean data through the MEGAHIT tool (v1.0.4-beta) and procured scaftigs devoid of N by segmenting the resultant scaffolds at the N junction.^82,83^ Utilizing MetaGeneMark, we anticipated ORFs for each sample’s scaftigs that exceeded 500 bp, eliminating details shorter than 100 nt in the predictions.^84^ To craft a non-redundant preliminary gene catalog, the CD-HIT software came in handy to curtail redundancy.^85^ Clean data from individual samples were synchronized with the starting gene catalog via Bowtie2, determining each sample’s gene read counts.^86^ For species annotation, sequences from Unigenes were aligned with known sequences from NCBI’s NR database, using the DIAMOND tool.^87^ Dimensionality reduction involved a blend of Krona, relative abundance summaries, and an abundance clustering heatmap, supplemented with PCA and NMDS. Between-group permutation tests at each taxonomic level were conducted using the Metastats method, from which we derived *p* values. These underwent correction using the Benjamini and Hochberg False Discovery Rate, producing *q* values. The LEfSe tool, with a default LDA Score of 3, facilitated LEfSe analysis. Species selection at the species gradient and RandomForest model construction was executed via Random Forest. For functional database annotations, Unigenes were synchronized with entries in the eggNOG and KEGG databases via the DIAMOND tool, cherry-picking the optimal Blast outcomes for ensuing examinations.^86,88^

### Detection of SCFAs

SCFAs like acetic, propionic, butyric, isobutyric, valeric, isovaleric, and caproic acid were identified using the MetWare platform. To prepare the serum for analysis, plasma or serum samples were first allowed to thaw, then agitated vigorously for 60 s. Fifty microliters of these samples were then followed by the addition of 100 μL of a 0.5% v/v phosphoric acid solution. This combination was shaken thoroughly for 180 s, after which 150 μL of MTBE solution was introduced. For muscle and bone tissue analysis, 50 mg of the tissue sample was meticulously measured and deposited into a 2 mL EP tube. To this, we added 0.2 mL of a 0.5% v/v phosphoric acid solution along with a miniature steel ball. This concoction was pulverized in three 15-s intervals, agitated for 600 s, and then subjected to ultrasonic waves for 300 s. Subsequently, 0.5 mL of MTBE solution was added. This mixture was again shaken for 180 s and ultrasonicated for an additional 300 s. The subsequent step involved centrifuging the blend at 12 000 r/min for 600 s at 4 °C. We extracted the supernatant for further GC–MS/MS examination, as outlined in prior methods.^89^ The GC–MS/MS analysis was performed using an Agilent 7890B gas chromatograph. This was equipped with a DB-FFAP column. Helium served as the carrier gas, maintaining a flow rate of 1.2 mL every minute. The substance was injected in a split mode, with the injected volume being 2 μL. The oven’s temperature was maintained at 90 °C for 60 s, then elevated to 100 °C at 25 °C every minute, further elevated to 150 °C at 20 °C every minute, stabilized for 36 s, hiked to 200 °C at 25 °C each minute, and stabilized for 30 s, spanning a total duration of 180 s. Each specimen underwent evaluation in a multiple reaction monitoring configuration.^90^

### Whole-limb grip strength test

We utilized a digital grip-strength meter (Columbus) to assess the limb grip strength of mice. As a preparatory step, mice were first familiarized with the testing environment for a duration of 10 min. Subsequently, each mouse was encouraged to grasp the metallic bar using its paws. Carefully, the tail of the mouse was drawn back until the mouse could no longer maintain its grip on the bar. The exerted force at the moment the mouse let go was documented as the maximum tension. This procedure was repeated for each mouse a total of ten times. The grip strength is then computed as the mean force, which can be adjusted according to the mouse’s body weight if necessary.

### Wire-hanging time test

Using its forelimbs, the mouse, when held by its tail, was encouraged to grip the wire’s center. If the mouse displayed undesirable actions such as trying to balance or leaping off, it was repositioned on the wire without pausing the stopwatch. The duration for which the mouse maintained its grip before letting go and falling was noted. If the mouse managed to cling for the test’s entire designated time, it was carefully taken off the wire, placed back in its enclosure, and the exact hang time was documented. If the mouse dropped before reaching the 600-s benchmark, it was given up to two additional attempts, ensuring a 1-min rest interval between each. The analysis considered the longest recorded hang duration from the trials.

### Treadmill exercise performance test

Following 2 days of acclimation, mice underwent a running performance test to assess their endurance. The animals were placed on a treadmill, where the speed increased by 5 cm/s every 2 min, with the incline set at 13%. Exhaustion was defined as the point when a mouse’s hindlimbs remained on the electric grid for over 10 s. The data were automatically recorded at the conclusion of the test, as previously described.^91^

### Creatine kinase (CK) activity test

Upon euthanasia, blood was drawn and allowed to clot by leaving it undisturbed at ambient temperature for half an hour. After clotting, the serum was isolated from the rest of the blood components by spinning it at a force of 1 000 *g* for a span of 10 min. This serum was then preserved at −80 °C until further analysis. We quantified the CK levels in the serum employing the Creatine Kinase Activity Assay Kit (MAK116, Sigma).

### Histology and immunofluorescence staining for muscle section

Entire muscle samples were swiftly encased in an O.C.T. compound and then flash-frozen, and then sliced into 10 μm sections. For the H&E technique, the sections underwent hematoxylin immersion for roughly 25 min, followed by a thrice rinse in tap water. Subsequently, they were submerged in Eosin for ~90 s, put through a dehydration process in ethanol grades and xylene, and then sealed with Permount for subsequent imaging. For immunofluorescence processes, fresh Gast muscles were set in O.C.T. and were flash-frozen using pre-chilled isopentane submerged in liquid nitrogen. Muscle sections of 10 μm thickness were prepared and kept at a temperature of −80 °C. To identify fiber types, these sections were first permeabilized, then treated with a solution of Triton X-100 and QuickBlock buffer (Beyotime, China) and left at ambient temperature for half an hour. Post this, an overnight incubation at 4 °C was done using a primary antibody mix containing anti-Dystrophin (ab15277, Abcam), anti-MyHC I (BA-D5, DSHB), anti-MyHC IIa (SC-71, DSHB), and anti-MyHC IIb (BF-F3, DSHB). After quadruple washes, a secondary antibody was mixed with Alexa Fluor variants 405, 488, 568 and 647, all appropriately diluted in QuickBlock secondary buffer (Beyotime, China), was applied for an hour at ambient temperature. All resulting visuals were documented using confocal laser scanning microscope. All the visual data quantifications, be it histological or immunofluorescence, were done using the ImageJ software tool (NIH, USA).

### Histology and immunofluorescence staining for bone section

For the immunofluorescence procedure on femurs, the samples were first extracted and then stabilized in 4% paraformaldehyde at 4 °C for a duration of 6 h. To achieve decalcification, we utilized 15% EDTA, following which the femurs were thoroughly rinsed and then submerged in a 30% sucrose solution. These specimens were then encased in OCT and sliced into 10 μm sections. The permeabilization step involved the use of 0.5% Triton X-100. Next, a blocking step was executed using diluted goat serum (1:10) for an hour, after which the sections were incubated with primary antibodies: OSX (1:100, sc-393325, Santa Cruz Biotechnology), OPN (1:100, AF7665, Beyotime), and OCN (1:100, GB11233, Servicebio). Then, secondary antibodies were employed and DAPI-containing medium was utilized for staining nuclei. For TRAP staining, paraffin-embedded sections of decalcified tibiae sections were baked 56 °C overnight. Deparaffinize and rehydrate sections: 2 × 10 min xylene (blot excess xylene before going into ethanol), 2 × 5 min 100% ethanol, 1 × 3 min 95% ethanol, 1 × 3 min 80% ethanol. Wash with TBST buffer 3 × 5 min (0.5% Tween PH 6.8). Put the TRAP staining buffer into the 37 °C incubator for 20 min. At the same time, put the sample into the 37 °C incubator 20 min. Drain the TBST buffer, staining samples in 37 °C incubator for 5–10 min. TRAP solution consisted of 100 mmol/L sodium acetate, 50 mmol/L sodium tartrate, naphthol AS-MX phosphate, and *N*,*N*-dimethyl formamide (MilliporeSigma). Images in the diaphysis of cortical bone were acquired using a microscope (Leica MZFLIII Microscope).

### Microcomputed tomography analysis

To assess bone density and structural intricacies, we employed the Inveon MM system (Siemens, Germany) for micro-CT. In our protocol, each specimen underwent scanning with settings as follows: pixel resolution at 8.89 μm, 60 kV voltage, 220 μA current, and a 1 500 ms exposure during each of the 360 rotational stages. The resultant images spanned 1 536 slices, with each voxel measuring 8.89 μm across every axis. Using two-dimensional images, we constructed three-dimensional (3D) visual representations. Subsequently, the Inveon Research Workplace (Siemens) was used to derive bone-related parameters: BMD, the ratio of BV/TV, the count of trabeculae (Tb. N), and Tb. Th within the femur’s area of interest.

### Biomechanical assays

To examine the femoral biomechanical properties, we utilized three-point bending assays. We engaged the femurs in a downward force at a consistent speed of 1.0 mm/min, using the servohydraulic tester (Instron 4302, Norwood, MA, USA), in line with established methods. Throughout the bending phase, the load-versus-deformation graphs were documented. We focused our analysis on the femur’s mid-diaphysis. Derived parameters included peak load, energy until ultimate load, Young’s modulus values, structural rigidity, and energy at fracture.

### Dynamic histomorphometric analyses

Ten days before euthanasia, mice received an intraperitoneal injection of calcein (Sigma, C0875). This was followed by an injection of alizarin-3-methyliminodiacetic acid (Sigma, A3882) 3 days before euthanasia. Following euthanasia, femurs were extracted, subjected to 80% ethanol for fixation, and subsequently dehydrated. The bones were then prepared to yield undecalcified sections. We employed BioQuant software (OSTEO, version v20.8.60, BioQuant) to calculate both the MAR and the bone formation rate relative to the bone surface (BFR/BS).

### Neutralization and supplementation experiments

To determine the roles of acetate and lactate in butyrate production by *C. butyricum*, the acids in the supernatant of *B. lactis* A6 cultures were neutralized. This neutralized supernatant was then used in subsequent experiments. The neutralized supernatant was supplemented with different concentrations of acetate and lactate as follows: neutralized supernatant, neutralized supernatant with low acetate concentration, neutralized supernatant with high acetate concentration, neutralized supernatant with low lactate concentration, neutralized supernatant with high lactate concentration, neutralized supernatant with acetate, neutralized supernatant with lactate, and neutralized supernatant with both acetate and lactate. The cultures were incubated anaerobically at 37 °C for 24 h, and the butyrate levels were measured as described above.

### Enzyme-linked immunosorbent assay (ELISA)

An enzyme-linked immunosorbent assay (ELISA) was performed to measure the levels of CTX (USCN LIFE, Wuhan EIAab Science Co., Ltd., Wuhan, China), PINP (Elabscience Biotechnology, Wuhan Elabscience Biotechnology Co. Ltd., Wuhan, China) and TNF-a, IL-6, IL-1β, and IL-17 (R&D systems, USA) in mice serum, according to the manufacturer’s protocol.

### Determination of serum FITC-dextran

Mice were first subjected to a 4-h fasting period on the day they were to be sacrificed. Subsequently, they received an intragastric dose of 150 μL FITC-dextran solution (80 mg/mL, 4 000 Da, Sigma-Aldrich) prepared in sterilized water. Three hours post-administration, blood was drawn from the mice’s orbital plexus. This blood was centrifuged at 3 000 × *g* at 4 °C to procure serum devoid of hemolysis. Using a fluorescence spectrophotometer set at an excitation of 485 nm and an emission of 528 nm, the FITC-dextran concentrations were measured.

### Isolation of T cells

T cells were isolated from bone marrow with a Pan T-cell Isolation Kit (Miltenyi Biotec, 130-095-130, Germany) according to the product manual. Briefly, bone marrow was flushed from femurs. All red blood cells were removed by RBC lysis buffer. After adjusting the number of cells, co-incubate the magnetic beads with the cells, and then pass cell-beads suspension through the sorting column on the magnetic rack. At this time, all T cells are adsorbed in the sorting column. Then, remove the magnetic rack and collect the T cells for subsequent experiments.

### Immunofluorescence staining for T cells

T cells of mice bone marrow were extracted by using a Pan T-cell Isolation Kit (Miltenyi Biotec) mentioned above, after washing these cells with PBS, T cells were fixed using 4% PFA at RT for 15 min. Centrifuge to remove the 4% PFA and wash again with PBS. Then, drop the cell suspension containing T cells onto a glass slide to make a cell smear and let it dry thoroughly. Subsequent operations are all carried out on glass slides. After cleaning with PBS, add QuickBlock™ Blocking Buffer for Immunol Staining (Beyotime, P0260, China) was used at room temperature for 1 h to reduce non-specific antibody binding in subsequent staining. After removing the Blocking Buffer, add the primary antibody, anti-NF-κB p65 antibody (ABCAM, ab32536, 1:200, UK), to slides, and incubate overnight at 4 °C. The next day, after removing the primary antibody, wash with PBS, add incubate with secondary antibody (Goat anti-Rabbit IgG H&L, ABCAM, ab150078, 1:500, UK) at RT in dark for 1 h. After removing the secondary antibody, clean with PBS and finally add Antifade Mounting Medium with DAPI (Beyotime, P0131) for sealing. Observe using confocal fluorescence microscopy.

### Western blot

Isolated T cells were lysed on ice for 30 min with RIPA buffer (Beyotime, Cat# P0013B) containing 10 mm protease inhibitor. Protein lysate was separated onto 10%–15% SDS–PAGE gels and then transferred to an Immobilon-PVDF membrane (Millipore, Billerica, MA). Membranes were blocked using 5% fat-free milk at 22 °C and subsequently incubated with primary antibodies at 4 °C overnight. Western blot analysis was performed with NF-κB signaling pathway associated primary antibody (Cell Signaling Technology, Danvers, MA, USA, dilution 1:1 000). After washing with TBST three times, the membranes were incubated with secondary peroxidase-conjugated antibodies for 1 h. Finally, the protein bands were visualized using an ECL detection kit (Millipore).

### FACS analysis of T cells

Bone marrow single-cell suspension is prepared as mentioned above. Briefly, bone marrow in femurs was flushed out and red blood cells were removed. Then, performed cell counting and extracted 2 million bone marrow cells for in vitro culture using RIPM1640 complete medium (contain 10% FBS and 1% B/S), while adding Cell Activation Cocktail (with Brefeldin A) (423303, Biolegend, USA) to stimulate cells to produce cytokines and block secretion at the same time for 16 h. The next day, collect cells and wash using Flow cytometry buffer. After staining the live/dead cell using Zombie Aquam Fixable Viability Kit (423102, Biolegend, USA) and the CD4-positive T cells with FITC anti-mouse CD4 (100406, Biolegend, USA), use Cyto-Fast™ Fix/Perm Buffer Set (426803, Biolegend, USA) for fixation and permeabilization at RT and add Brilliant Violet 421™ anti-mouse IL-17A (506926, Biolegend, USA) and APC anti-mouse IFN-γ (505810, Biolegend, USA) to stain the intracellular marker. And then, flow cytometry analysis was done on BD FACSAria III (BD, USA).

### Statistical analysis

Data analysis was conducted using the software GraphPad Prism, version 8.4.3 (GraphPad Software, CA, USA). Values are expressed as mean ± standard deviation (SD). For comparisons, we employed either the two-tailed Welch’s *t*-test or two-way ANOVA. A *P* value < 0.05 indicated statistical significance.

## Supplementary information

Supplementary Information

WB-Unprocessed data

## Data Availability

All data are available from the authors upon reasonable request.
