# Supplementary material for: Bifidobacterium animalis subsp. lactis A6 ameliorates bone and muscle loss via modulating gut microbiota composition and enhancing butyrate production
Source: Bone Res. 2025 Feb 25;13:28. doi: 10.1038/s41413-024-00381-1 (PMC11862215; doi:10.1038/s41413-024-00381-1)
Supplement: Supplementary file 1 — Supplementary Information [file 41413_2024_381_MOESM1_ESM.doc]

**Supplemental Information**

***Bifidobacterium animalis* subsp. *lactis* A6 ameliorates bone and muscle loss via modulating gut microbiota composition and enhancing butyrate production**

Ming Chen^1,2#^, Yi Li^1,2#^, Zhengyuan Zhai^3^, Hui Wang^3^, Yuan Lin^4^, Feifan Chang^1,2^, Siliang Ge^1,2^, Xinyu Sun^1,2^, Wei Wei^5^, Duanyang Wang^4^, Mingming Zhang^1,2^, Ruijing Chen^1,2^, Haikuan Yu^1,2^, Taojin Feng^1,2^, Xiang Huang^1,2^, Dongliang Cheng^1,2^, Jiang Liu^4^, Wenxuan Di^3^, Yanling Hao^3*^, Pengbin Yin^1,2*^, Peifu Tang^1,2^

^1^Senior Department of Orthopedics, The Fourth Medical Center of Chinese PLA General Hospital, Beijing, China

^2^ National Clinical Research Center for Orthopedics, Sports Medicine & Rehabilitation, Beijing, China

^3^ Key Laboratory of Precision Nutrition and Food Quality, Department of Nutrition and Health, China Agricultural University, Beijing, China

^4^ The Department of Orthopedic Surgery, Second Affiliated Hospital of Harbin Medical University, Harbin, China

^5^ Department of Clinical Nutrition, Peking Union Medical College Hospital, Chinese Academy of Medical Science and Peking Union Medical College, Beijing, China

Correspondence:

Pengbin Yin, PhD

Professor, Department of Orthopedics, Chinese PLA General Hospital

Professor, National Clinical Research Center for Orthopedics, Sports Medicine & Rehabilitation

No. 28 Fuxing Road, Beijing, 100853, P. R. China

Tel: 86-10-66938201, Fax: 86-10-6821-2342, E-mail: [yinpengbin@gmail.com](mailto:yinpengbin@gmail.com)

Yanling Hao, PhD

Director, Key Laboratory of Precision Nutrition and Food Quality, Department of Nutrition and Health, China Agricultural University, Beijing, China

No.17 Qing Hua East Road, Beijing, 100083, P. R. China

E-mail: haoyl@cau.edu.cn

^#^ These authors contributed equally to this work.


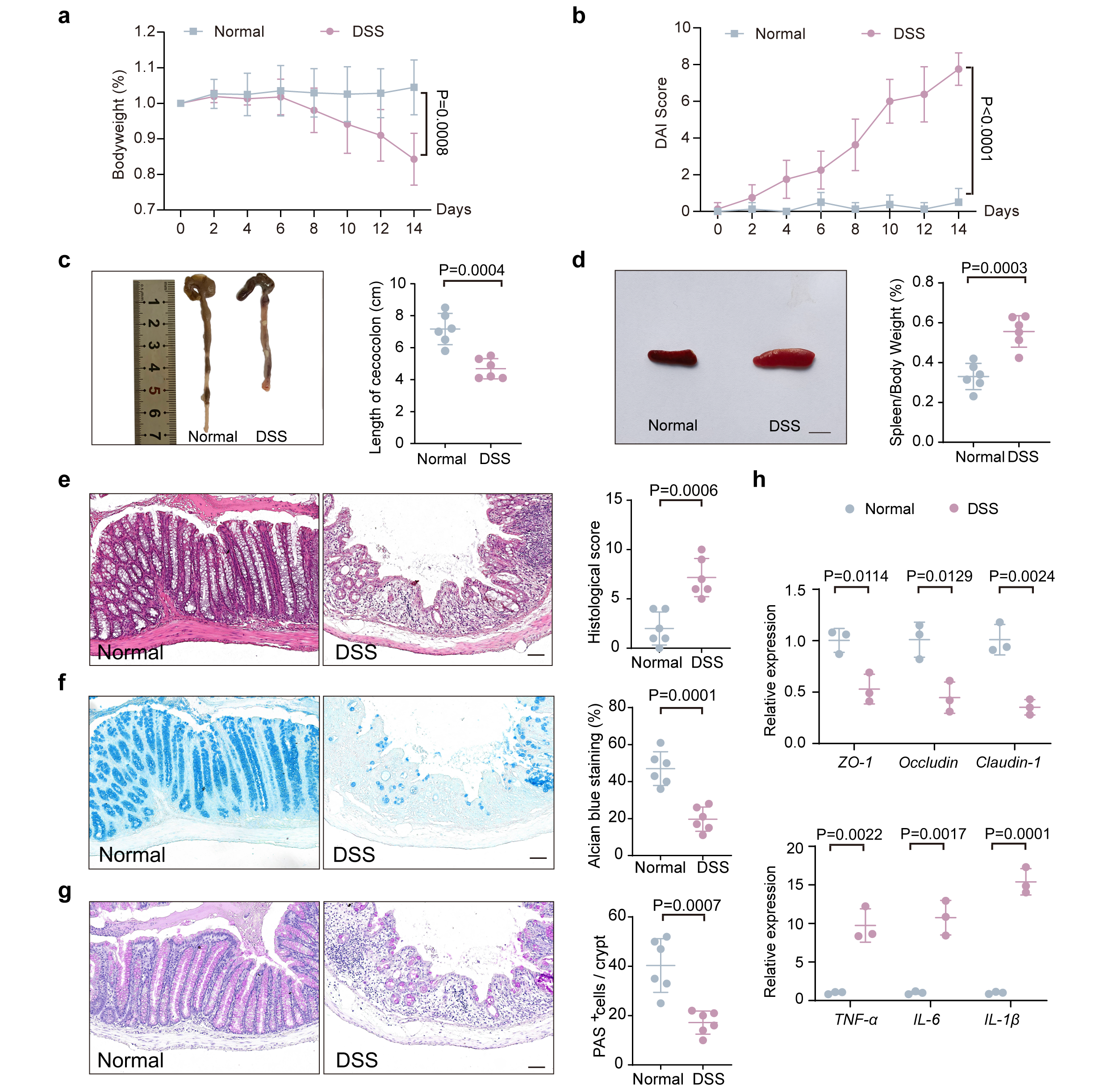


**Fig. S1** Intestine damage is induced after dextran sodium sulfate (DSS) exposure. **a** Body weight change during the 14-day phase of DSS exposure (n=6). **b** DAI score evaluation during the 14-day phase of DSS exposure (n=6). **c** Representative images and quantitative analyses of colon tissues (n=6). **d** Images and quantification of spleen tissues (n=6). Scale bar, 1cm. **e-g** Representative images and quantification analysis of Hematoxylin and Eosin (H&E) (e), Alcian Blue (AB) (f) and Periodic Acid Schiff (PAS) staining (g) from each group (n=6). Scale bar, 50μm. **h** mRNA expressions of genes for tight junctions (*ZO-1*, *Occludin*, and *Claudin-1*) and inflammatory indicators (*TNF-α*, *IL-6*, and *IL-1β*) in colon tissues, tested by qPCR (n=3). Values are represented as the average ± standard deviation. Significance (p value) is calculated using two-way ANOVA multiple comparisons (a,b) or two-tailed Welch’s t test (c-h).

**
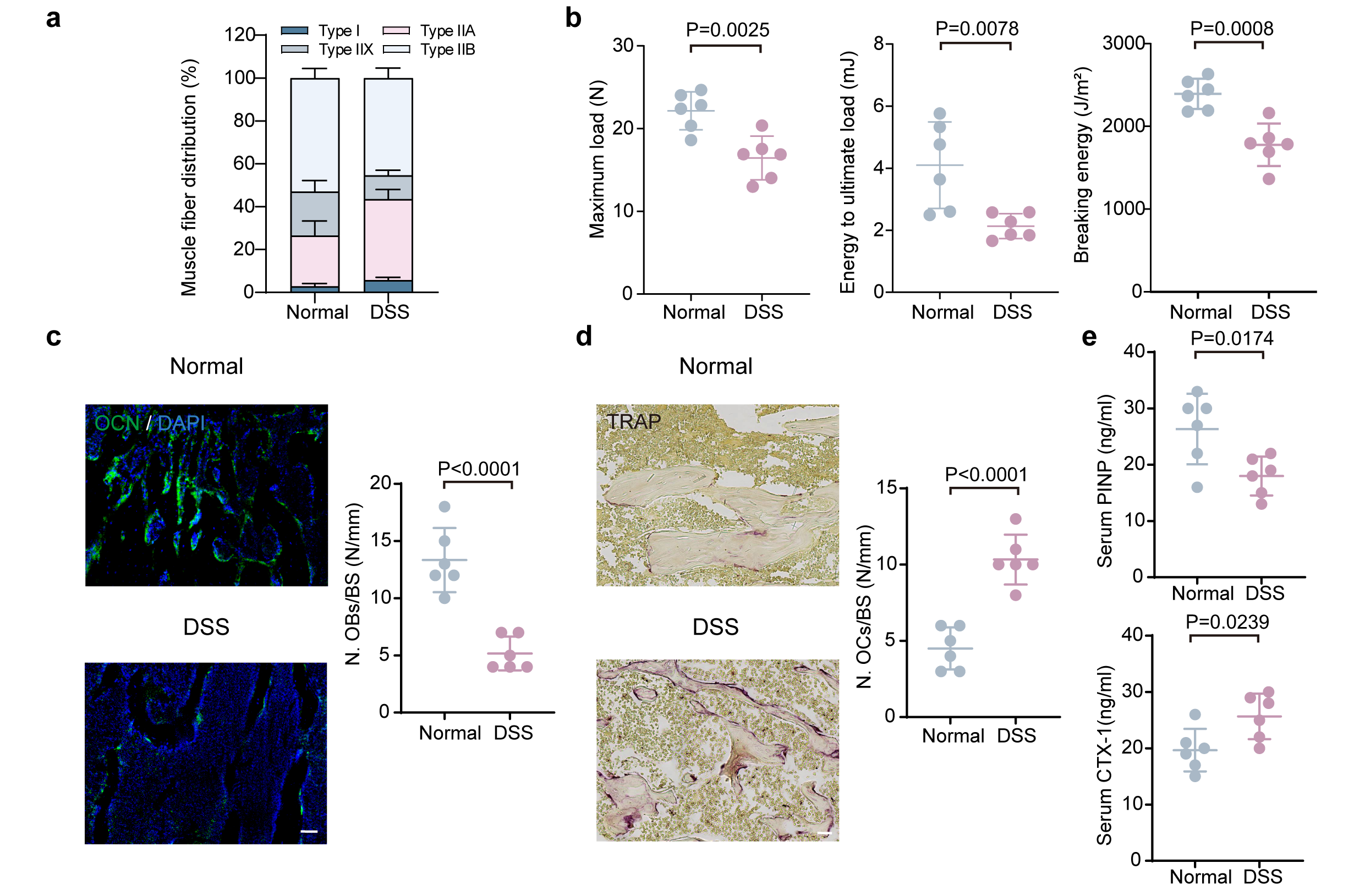
**

**Fig. S2** Analysis of muscle and bone phenotypes after DSS exposure. **a** Stacked bar graphs visualizing muscle fiber type compositions. **b** Biomechanical analysis of femur from mice. The parameters measured include the maximum load, energy to ultimate load and breaking energy (n=6). **c** Representative OCN-stained sections with quantification of the number of osteoblasts (N. OBs) on trabecular bone surface (BS) in distal femora from mice (n=6). Scale bar, 50μm. **d** Representative TRAP-stained sections with quantitation of the number of osteoclasts (N. OCs) (n=6). Scale bar, 30μm. **e** ELISA for serum PINP and CTX-1 (n=6). Values are represented as the average ± standard deviation. The significance level (p value) was determined through a two-sided Welch’s t-test.


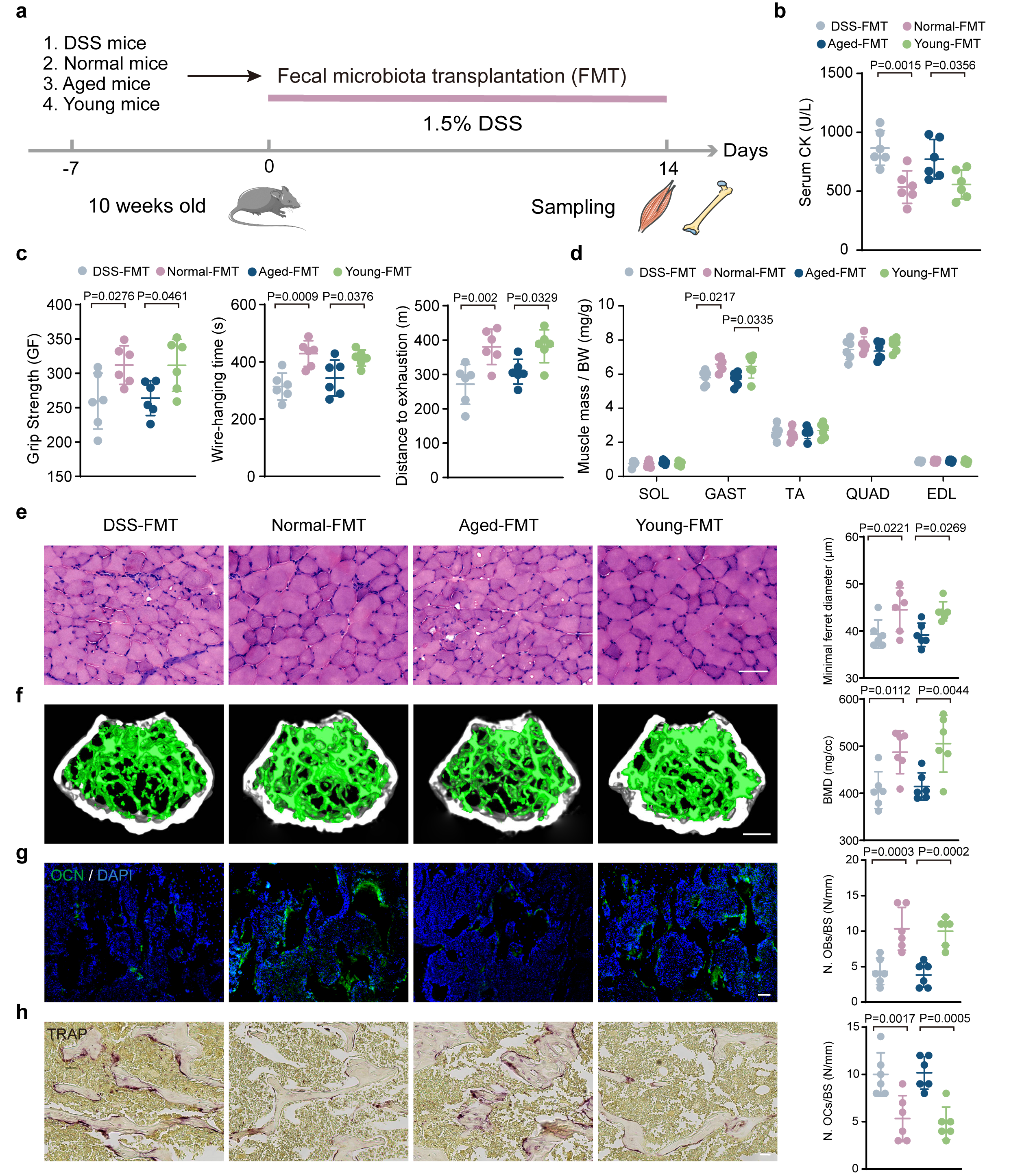


**Fig. S3** Fecal microbiota transplantation (FMT) reveals different impacts of microbiota on bone and skeletal muscle health. **a** Schematic representation illustrating the design. Mice were divided into four groups: Fecal microbiota from DSS, normal, aged and young mice were respectively transplanted into DSS-induced mice for 2 weeks. **b** Measurement of CK activity of the serum (n=6). **c** Assessment of physical performance using all-limb force, longest suspension time, and distance to exhaustion evaluated by handgrip, hanging wire tests, and treadmill, respectively (n=6). **d** Muscle mass analysis from each group (n=6). **e** Representative images of H&E staining in gastrocnemius cross-sections and quantification of average minimal Feret’s diameter of myofibers (n=6). Scale bar, 50μm. **f** Representative micro-CT images of distal femoral metaphyseal trabecular bone and quantitative analysis of BMD (n=6). Scale bar, 500μm. **g** Representative OCN-stained sections with quantification of N. OBs in distal femora from mice (n=6). Scale bar, 50μm. **h** Representative TRAP-stained sections with quantitation of N. OCs (n=6). Scale bar, 30μm. Values are represented as the average ± standard deviation. The significance level (p value) was assessed with one-way ANOVA.


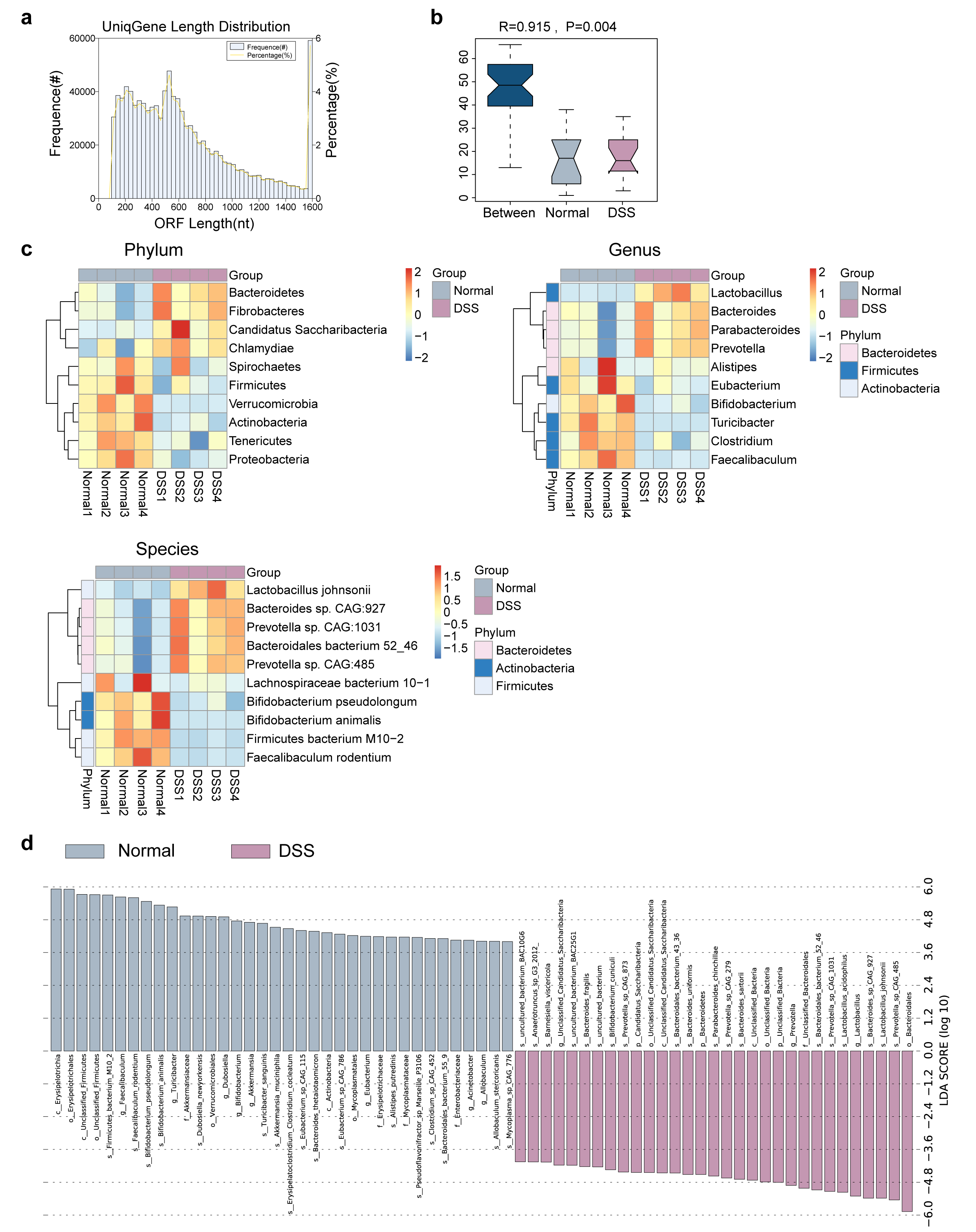


**Fig. S4** Analysis of gut microbiota composition after DSS exposure in mice. **a** Statistical analysis of gene catalogue length distribution. **b** Anosim analysis based on phylum level between the two groups. **c** Representative heatmaps of top 10 relative fecal bacterial abundances in phylum, genus and species level, respectively. **d** Representative images of LDA score analysis across different taxa levels.


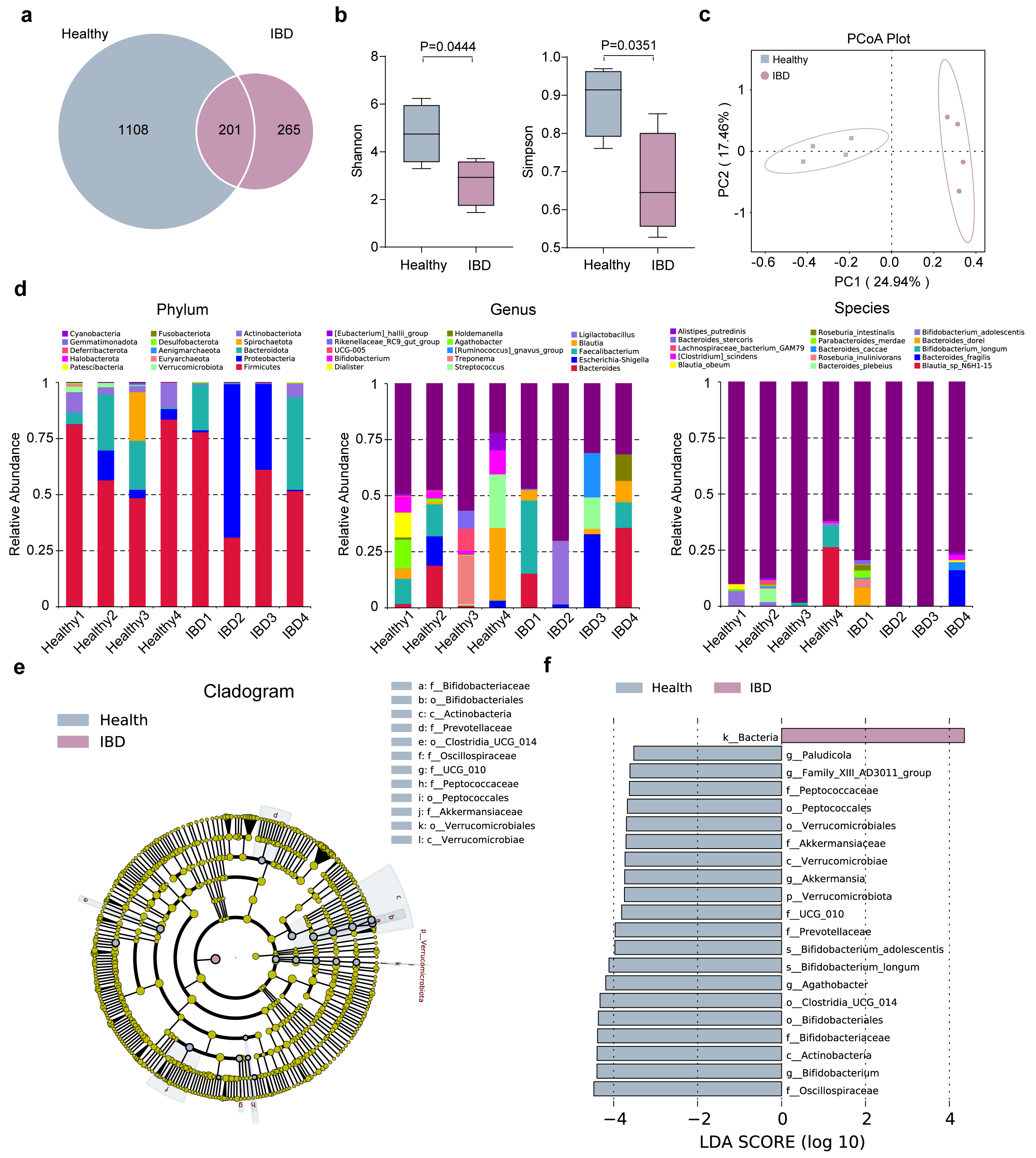


**Fig. S5** Analysis of gut microbiota composition in healthy individuals and IBD patients. **a** Venn diagram analysis of gene numbers detected in two groups. **b** The box plot illustrates *α*-Diversity using the Shannon and Simpson indices. **c** Principal Coordinate Analysis (PCoA) of *β*-diversity at the phylum tier is conducted via a Bray-Curtis matrix comparison for both groups. **d** Structure plot of the relative fecal bacterial abundances in phylum and genus-level based on Bray-Curtis distance. **e, f** Analysis of Cladogram generated from LEfSe (e) and LDA score (f) across different taxa levels.

**
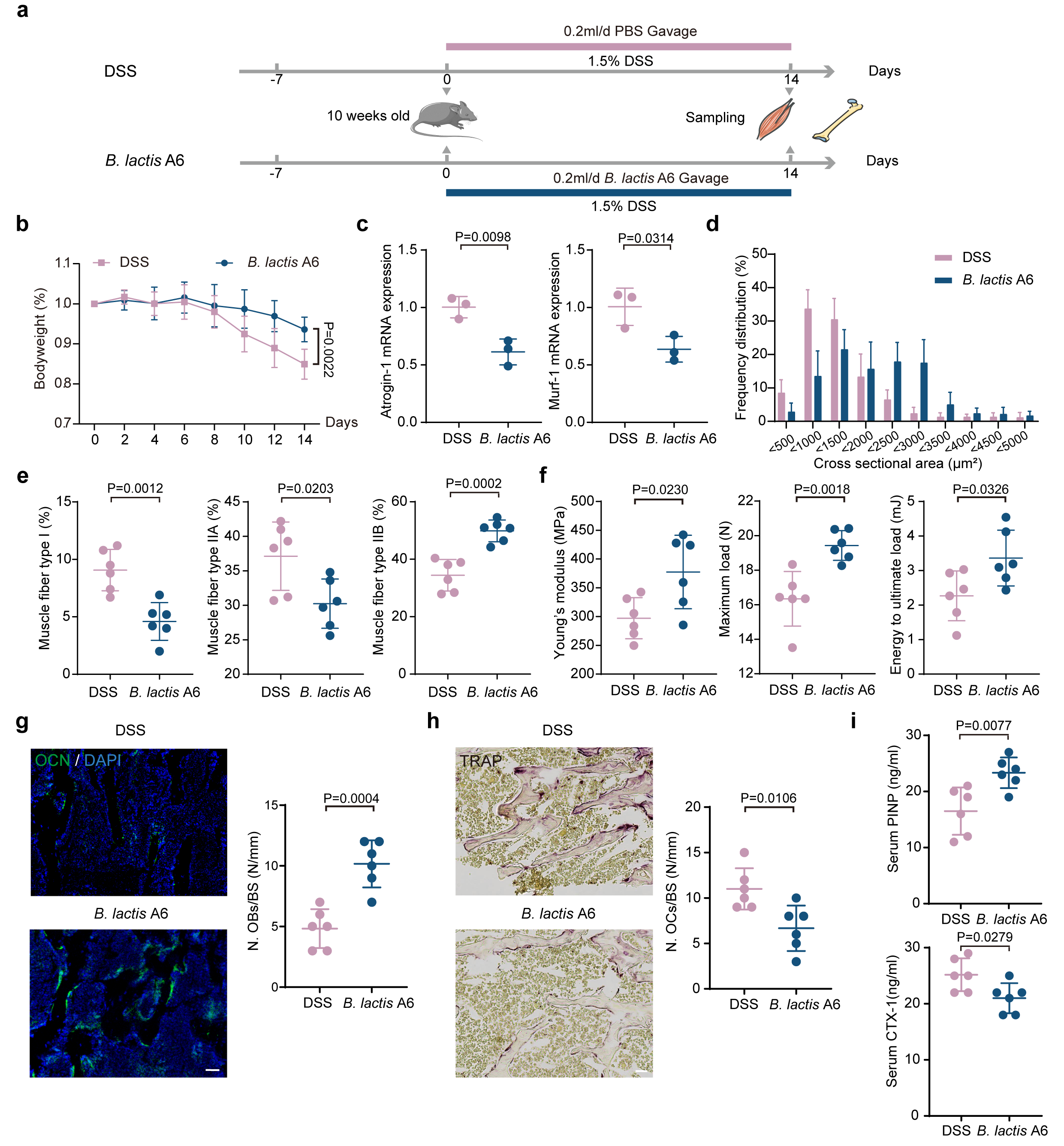
**

**Fig. S6** Analysis of muscle and bone phenotypes after *B. lactis* A6 supplementation. **a** Schematic representation illustrating the design. Mice were divided into two groups, and received a daily oral gavage of DSS (DSS group) or *B. lactis* A6 (DSS+ *B. lactis* A6 group) for 2 weeks. **b** Body weight change during the 14-day phase of DSS exposure and *B. lactis* A6 intervention (n=6). **c** mRNA Expressions of *Atrogin-1* and *Murf-1* in gastrocnemius, tested by qPCR (n=3). **d** Frequency distribution of myofiber CSA. **e** Muscle fiber type quantification (n=6). **f** Biomechanical analysis of femur from mice (n=6). **g** Representative OCN-stained sections with quantification of N. OBs in distal femora from mice (n=6). Scale bar, 50μm. **h** Representative TRAP-stained sections with quantitation of N. OCs (n=6). Scale bar, 30μm. **i** ELISA for serum PINP and CTX-1 (n=6). Values are represented as the average ± standard deviation. Significance (p value) is calculated using two-way ANOVA multiple comparisons (b) or two-tailed Welch’s t test (c-i).

**
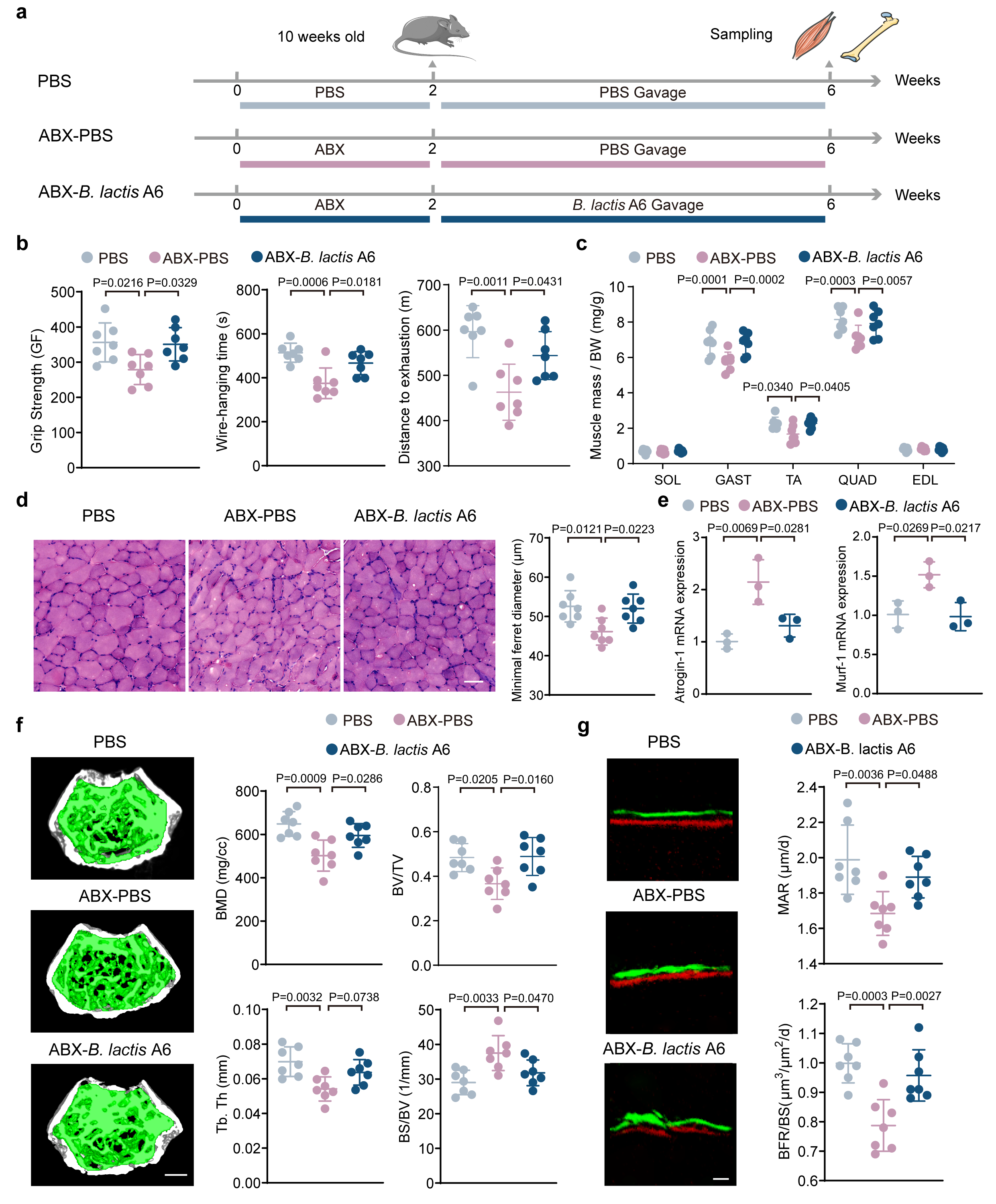
**

**Fig. S7** *B. lactis* A6 supplementation alleviates bone and muscle loss in wide-spectrum antibiotics (ABX)-treated mice. **a** Schematic representation illustrating the design. Mice were divided into three groups: the PBS group received oral PBS for 2 weeks followed by 4 weeks of PBS; the ABX-PBS group underwent 2 weeks of broad-spectrum antibiotics (ABX), followed by 4 weeks of PBS; and the ABX-B. lactis A6 group received 2 weeks of ABX followed by 4 weeks of *B. lactis* A6 probiotics. **b** Assessment of physical performance using all-limb force, longest suspension time, and distance to exhaustion evaluated by handgrip, hanging wire tests, and treadmill, respectively (n=7). **c** Muscle mass analysis from each group (n=7). **d** Representative images of H&E staining in gastrocnemius cross-sections and quantification of average minimal Feret’s diameter of myofibers (n=7). Scale bar, 50μm. **e** mRNA Expressions of *Atrogin-1* and *Murf-1* in gastrocnemius, tested by qPCR (n=3). **f** Representative micro-CT images of distal femoral metaphyseal trabecular bone. Quantitative analysis of bone mass, including BMD, BV/TV, Tb. Th and BS/BV (n=7). Scale bar, 500μm. **g** Representative images and quantification of new bone formation assessed by dynamic histomorphometric analyses (n=7). Scale bar, 25μm. Values are represented as the average ± standard deviation. The significance level (p value) was assessed with one-way ANOVA.

**
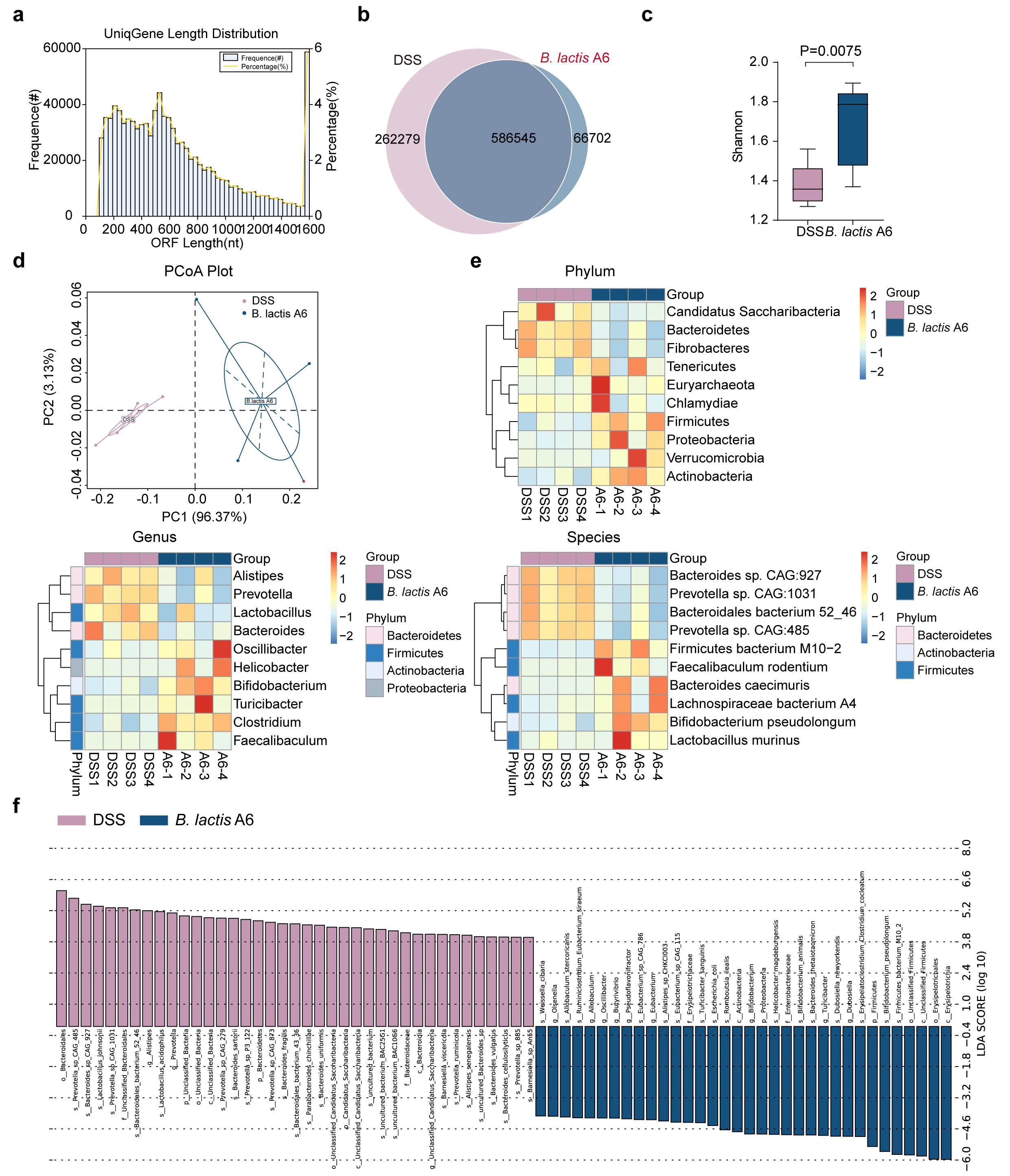
**

**Fig. S8** The impacts of *B. lactis* A6 on intestinal microbiota composition. **a** Statistical analysis of gene catalogue length distribution. **b** Venn diagram analysis of gene numbers detected in two groups. **c** The Shannon indices illustrate the *α*-diversity through a box plot depiction. **d** Principal Coordinate Analysis (PCoA) showcases *β*-diversity at the phylum level, utilizing a Bray-Curtis matrix to compare both groups. **e** Representative heatmaps of top 10 relative fecal bacterial abundances in phylum, genus and species level, respectively. **f** Representative images of LDA score analysis across different taxa levels.

**
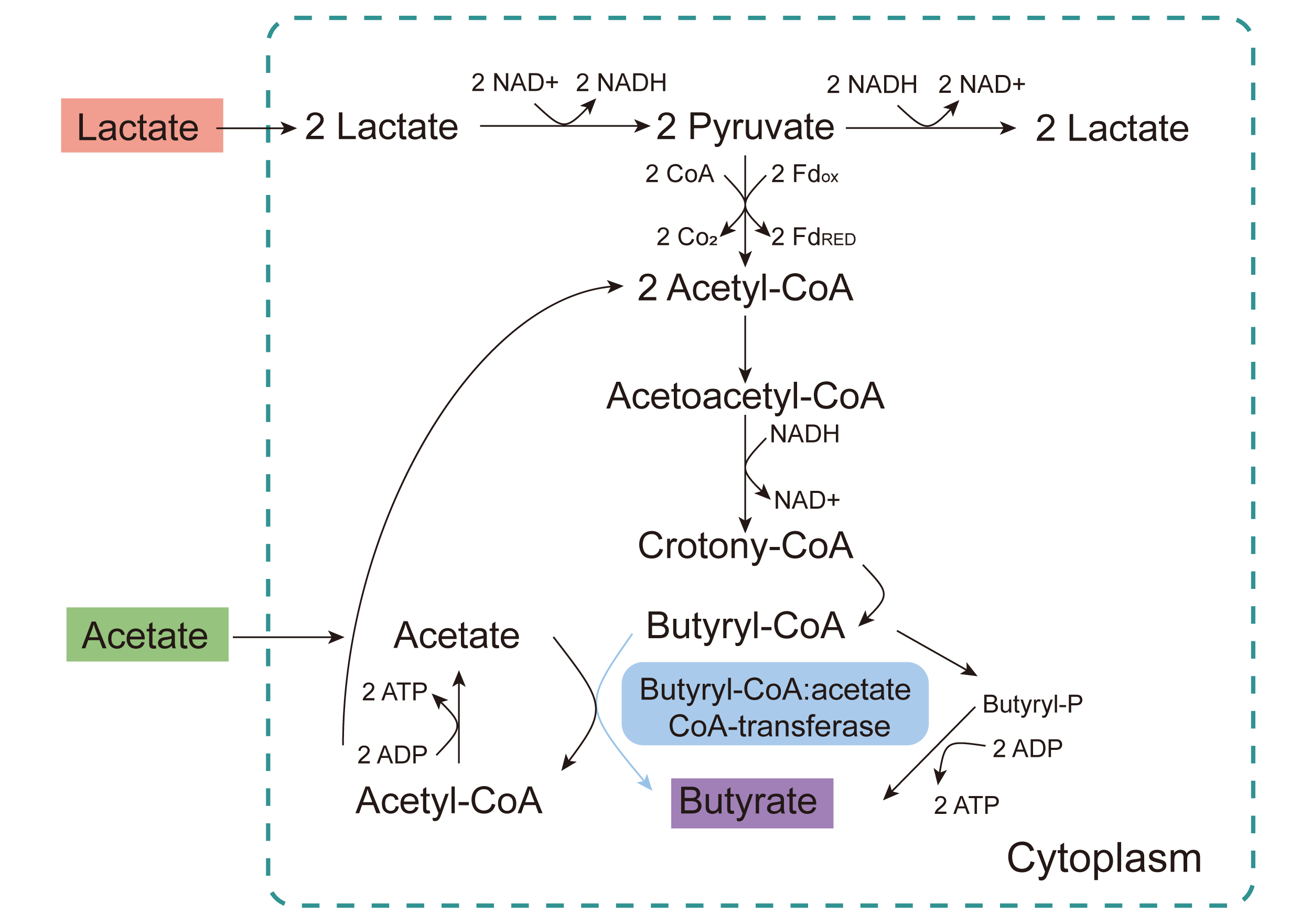
**

**Fig. S9** Schematic representation of butyrate pathway.


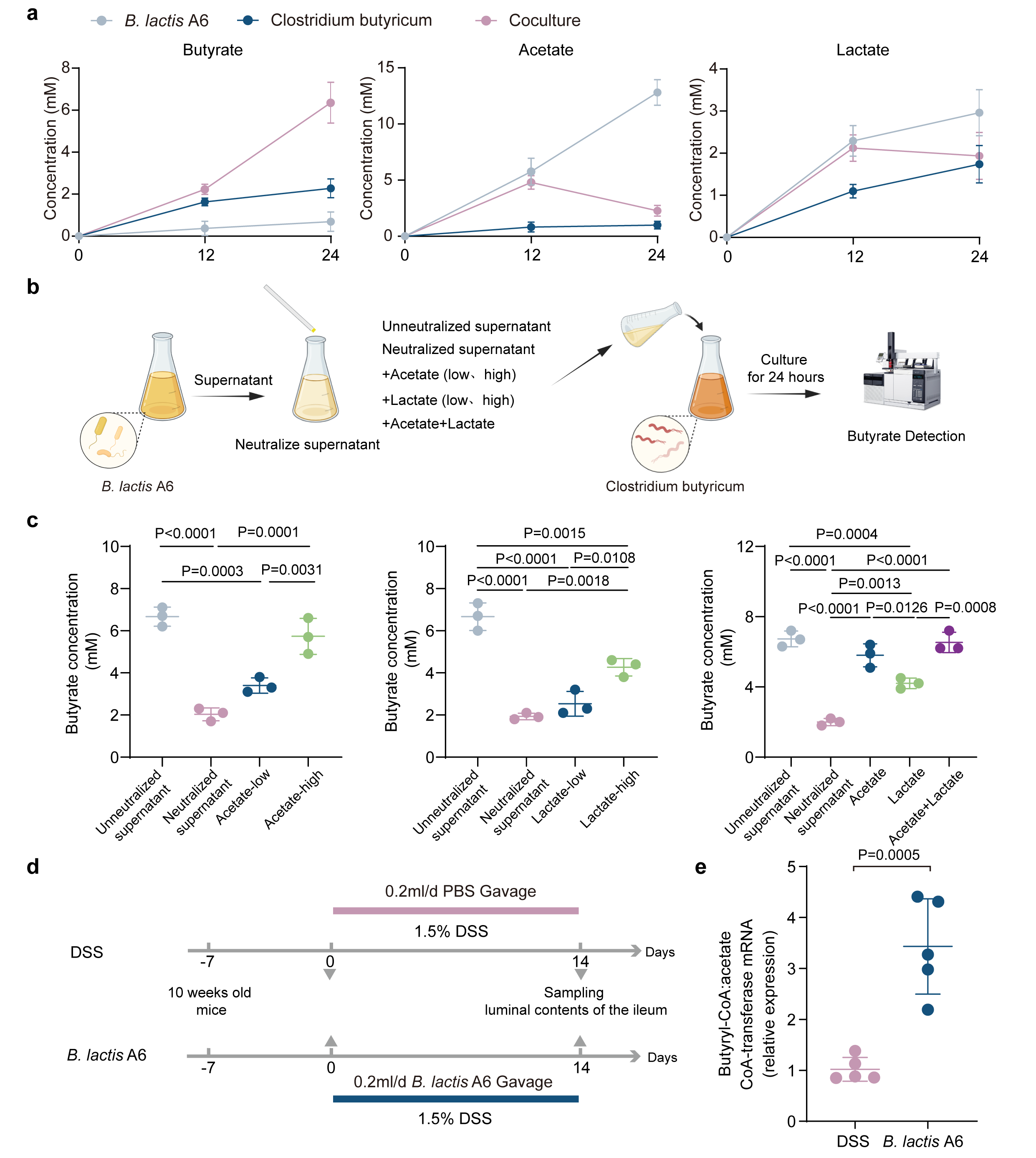


**Fig. S10** Cross-feeding mechanism and butyrate production enhancement by *B. lactis* A6. **a** Levels of butyrate, acetate, and lactate were measured in cultures of *B. lactis* A6, *Clostridium butyricum*, and their co-culture at 0, 12, and 24 hours (n=3). **b** Schematic representation of the experimental design for neutralization and supplementation experiments. The supernatant from *B. lactis* A6 cultures was neutralized to pH 7.0 and supplemented with different concentrations of acetate and lactate before being added to *C. butyricum* cultures. Butyrate production was measured after 24 hours of anaerobic incubation at 37°C. **c** Quantification of butyrate levels in *C. butyricum* cultures (n=3). **d** Experimental design for the in vivo administration of *B. lactis* A6. Luminal contents of the ileum were collected for analysis after 14 days treatment. **e** Measurement of transcript levels of butyryl-CoA:acetate CoA-transferase in the luminal contents of the ileum in mice administered *B. lactis* A6 or vehicle control (n=5). Values are represented as the average ± standard deviation. Significance (p value) is calculated using one-way ANOVA (c) or two-tailed Welch’s t test (e).

**
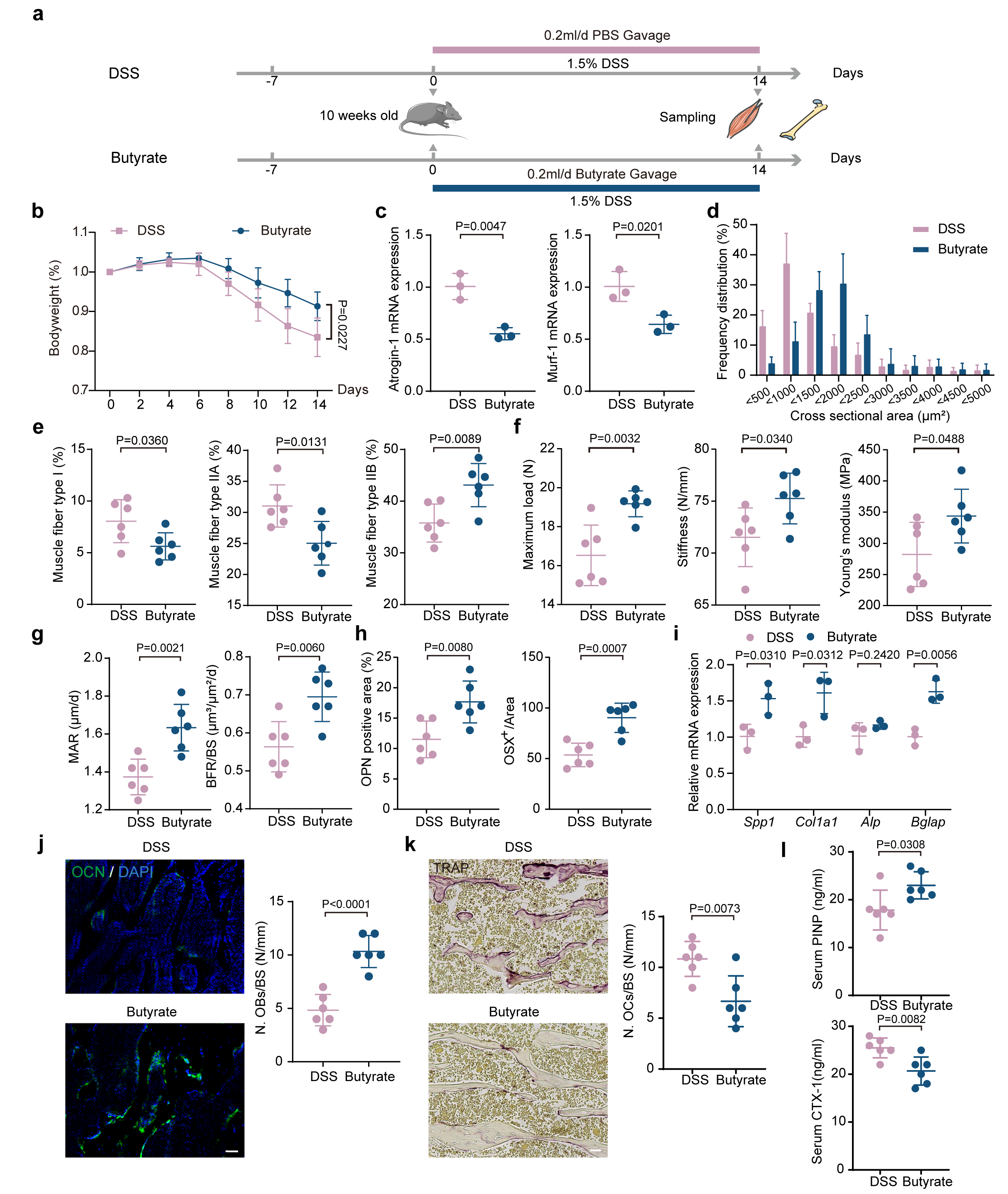
**

**Fig. S11** Analysis of muscle and bone phenotypes after butyrate administration. **a** Schematic representation illustrating the design. Mice were divided into two groups, and received a daily oral gavage of DSS (DSS group) or butyrate (DSS+ butyrate group) for 2 weeks. **b** Body weight change during the 14-day phase of DSS exposure and butyrate supplementation (n=6). **c** mRNA Expressions of *Atrogin-1* and *Murf-1* in gastrocnemius, tested by qPCR (n=3). **d** Frequency distribution of myofiber CSA. **e** Muscle fiber type quantification. **f** Biomechanical analysis of femur from mice (n=6). **g** Quantitative analysis of MAR and BFR/BS (n=6). **h** Quantification analysis for immunofluorescence staining of OPN and OSX in femur tissues (n=6). **i** mRNA Expressions of osteogenesis gene (*Spp1*, *Col1a1*, *Alp* and *Bglap*) expression in tibia, tested by qPCR (n=6). **j** Representative OCN-stained sections with quantification of N. OBs in distal femora from mice (n=6). Scale bar, 50μm. **k** Representative TRAP-stained sections with quantitation of N. OCs (n=6). Scale bar, 30μm. **l** ELISA for serum PINP and CTX-1 (n=6). Values are represented as the average ± standard deviation. Significance (p value) is calculated using two-way ANOVA multiple comparisons (b) or two-tailed Welch’s t test (c-l).

**
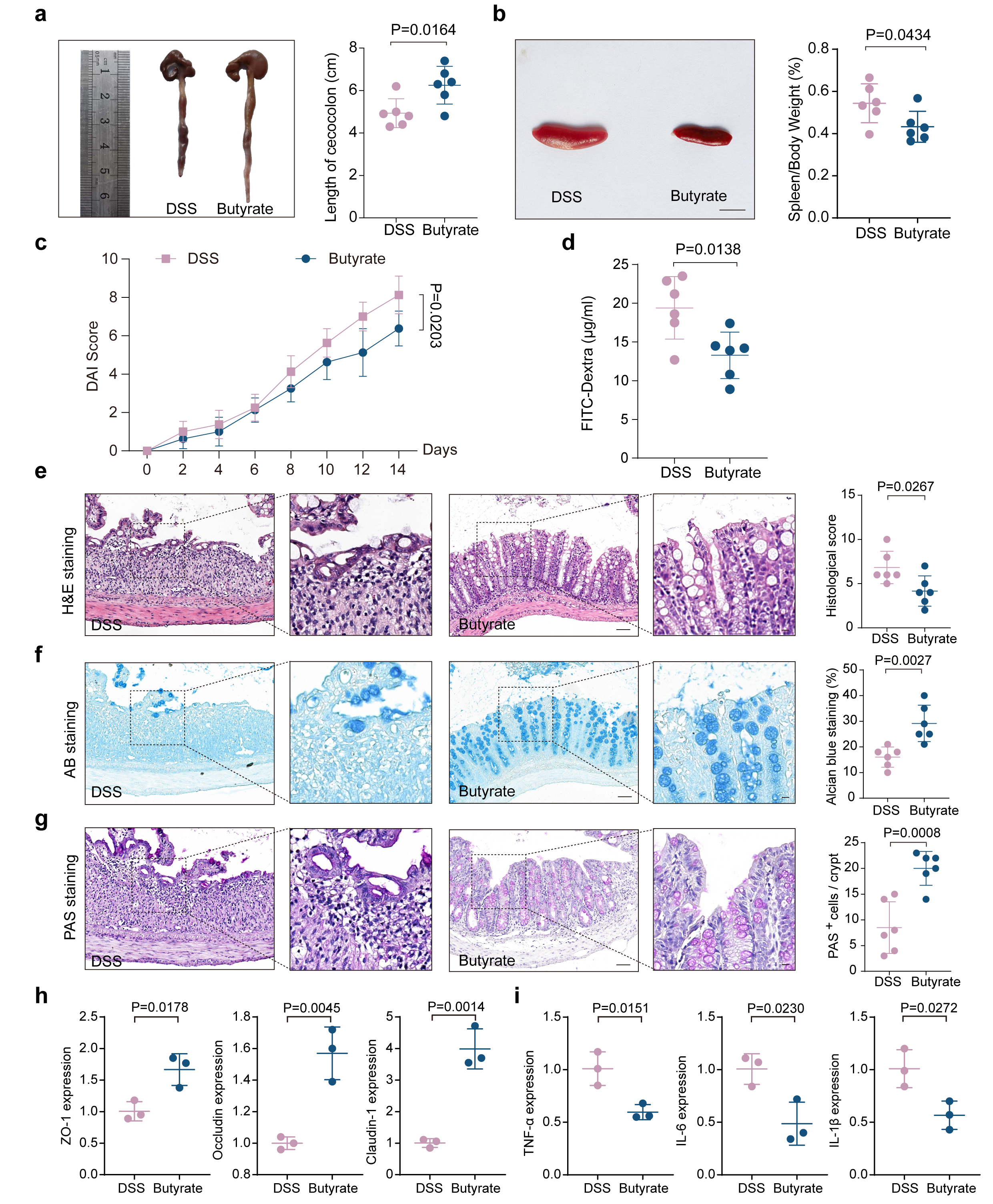
**

**Fig. S12** Butyrate ameliorates bone and muscle loss by preserving gut barrier function. **a** Images and quantification of colon tissues (n=6). **b** Images and quantification of spleen tissues (n=6). Scale bar, 1cm. **c** DAI score evaluation during the 14-day phase of DSS exposure and butyrate supplementation (n=6). **d** FITC-dextran concentrations in serum (n=6). **e-g** Representative images and quantification analysis of H&E staining (e), AB staining (f) and PAS staining (g) from each group (n=6). Scale bar, 50μm and 10μm, respectively. **h, i** mRNA expressions for *ZO-1*, *Occludin*, and *Claudin-1* (h) and *TNF-α*, *IL-6*, and *IL-1β* (i) in colon tissues, tested by qPCR (n=3). Values are represented as the average ± standard deviation. Significance (p value) is calculated using two-way ANOVA multiple comparisons (c) or two-tailed Welch’s t test (a, b, d-i).

**
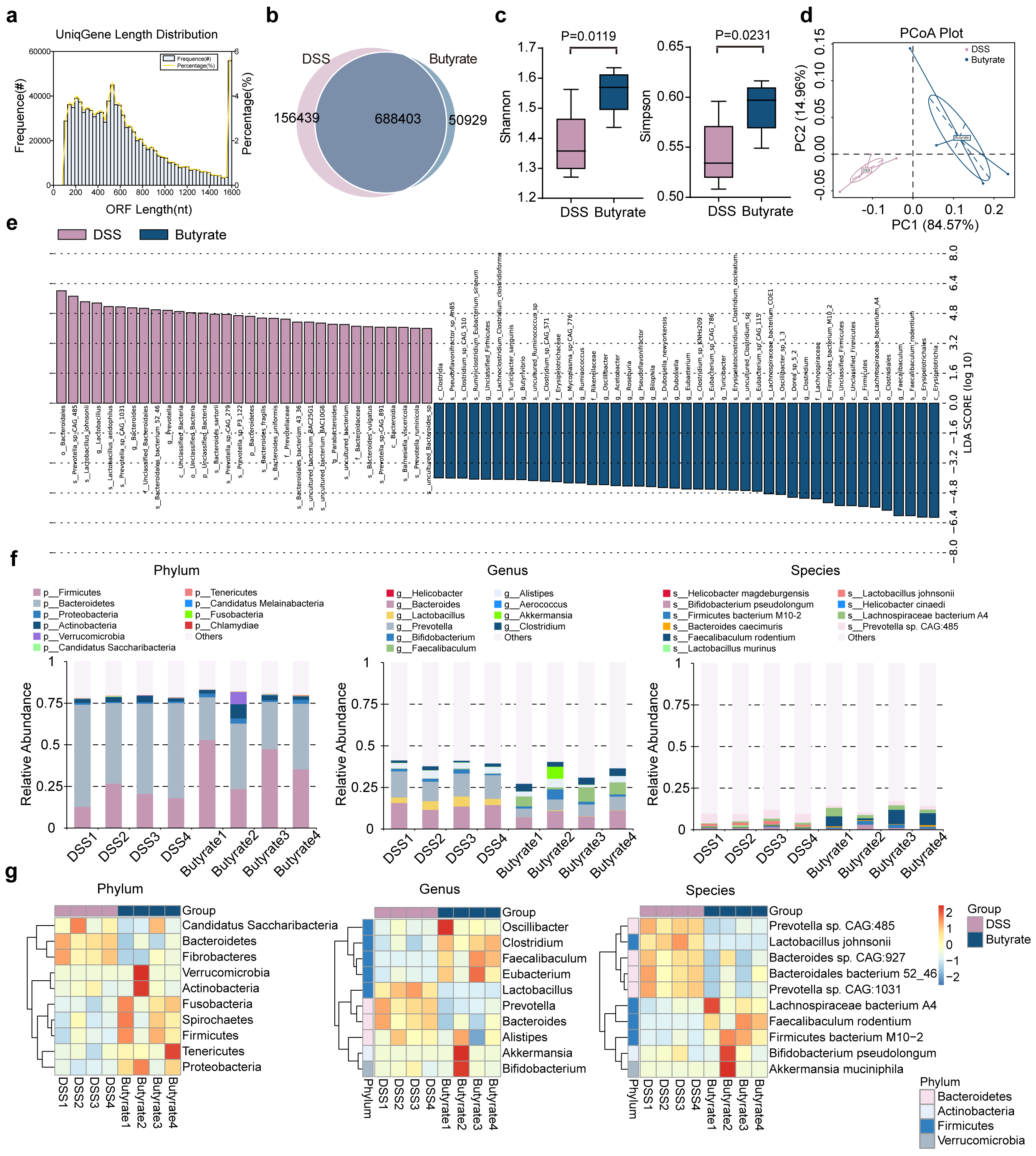
**

**Fig. S13** The effects of butyrate treatment on intestinal microbiota composition. **a** Statistical analysis of gene catalogue length distribution. **b** Venn diagram analysis of gene numbers detected in two groups. **c** The box plot showcases *α*-Diversity, indicating by the Shannon and Simpson metrics. **d** A principal coordinates analysis (PCoA) of *β*-diversity, grounded on the taxonomy at the phylum tier, utilizes the Bray-Curtis metric to compare the two groups. **e** Representative images of LDA score analysis across different taxa levels. **f** Structure plot of the relative fecal bacterial abundances in phylum, genus and species level based on Bray-Curtis distance. **g** Representative heatmaps of top 10 relative fecal bacterial abundances in phylum, genus and species level, respectively.

**Table S1**. Disease activity index scoring

| **Score** | **Body weight loss (%)** | **Stool consistency** | **Stool occult blood ^a^** |
| --- | --- | --- | --- |
| 0 | None | Normal | Negative |
| 1 | 1-5 | Soft stools | Positive (+) |
| 2 | 6-10 | Very soft stools | Positive (++) |
| 3 | 11-18 | Watery stools (Diarrhea) | Positive (+++) |
| 4 | ＞18 |  | Visible rectal bleeding |

^a^ Positive (+) indicates color change from light green to green within 10s, while Positive (++) show color change from green to blue within 30s and Positive (+++) to dark blue immediately according to the instructions of manufacturer (Brybio, Beijing, China).

**Table S2**. Histological scores of colon damage

| **Score** | **Inflammation severity** | **Inflammation extent** | **Crypt damage** |
| --- | --- | --- | --- |
| 0 | None | None | None |
| 1 | Mild | Mucosa | Basal 1/3 damaged |
| 2 | Moderate | Mucosa and submucosa | Basal 2/3 damaged |
| 3 | Severe | Transmural | Crypt lost, surface epithelium  present |
| 4 | / | / | Crypt and surface epithelium lost |

**Table S3**. RT-qPCR primer list

| **qPCR Primer list** | **Forward Primer** | **Reverse Primer** |
| --- | --- | --- |
| *ZO-1* | GGCCTTGGCCTAGCATACAC | GTCTTCATTTGACCCTCCCTC |
| *Occludin* | TCACTTTTCCTGCGGTGACTT | GGGAACGTGGCCGATATAAT |
| *Claudin-1* | AGCTGTGCATGGCCTCTTGT | CCAATGTCAATGGCAACACCC |
| *TNF-α* | GGTGCCTATGTCTCAGCCTCTT | GCCATAGAACTGATGAGAGGGAG |
| *IL-6* | TACCACTTCACAAGTCGGAGGC | CTGCAAGTGCATCATCGTTGTTC |
| *IL-1β* | TGGACCTTCCAGGATGAGGACA | GTTCATCTCGGAGCCTGTAGTG |
| *Atrogin-1* | CTTCTCGACTGCCATCCTGGAT | TCTTTTGGGCGATGCCACTCAG |
| *Murf-1* | TACCAAGCCTGTGGTCATCCTG | ACGGAAACGACCTCCAGACATG |
| *Spp1* | GCTTGGCTTATGGACTGAGGTC | CCTTAGACTCACCGCTCTTCATG |
| *Col1a1* | CCTCAGGGTATTGCTGGACAAC | CAGAAGGACCTTGTTTGCCAGG |
| *Alp* | CCAGAAAGACACCTTGACTGTGG | TCTTGTCCGTGTCGCTCACCAT |
| *Bglap* | GCAATAAGGTAGTGAACAGACTCC | CCATAGATGCGTTTGTAGGCGG |
| *β-actin* | CATTGCTGACAGGATGCAGAAGG | TGCTGGAAGGTGGACAGTGAGG |

Table S4. Quantitative concentrations of SCFAs after DSS exposure

|  | **Serum (ng/ml)** | | **P value** | **Muscle (μg/g)** | | **P value** | **Bone (μg/g)** | | **P value** |
| --- | --- | --- | --- | --- | --- | --- | --- | --- | --- |
|  | **Normal** | **DSS** |  | **Normal** | **DSS** |  | **Normal** | **DSS** |  |
| AA | 1017±194.2 | 643.4±229.1 | 0.0034 | 34.90±10.27 | 26.71±10.27 | 0.1176 | 2.985±0.723 | 2.237±0.687 | 0.0521 |
| PA | 155.1±77.59 | 147.9±43.27 | 0.8205 | 0.442±0.118 | 0.323±0.098 | 0.0461 | 0.555±0.271 | 0.342±0.077 | 0.0505 |
| IBA | 448.1±104.5 | 321.3±65.61 | 0.0115 | 2.060±0.694 | 1.714±0.365 | 0.2320 | 0.142±0.098 | 0.089±0.046 | 0.1930 |
| BA | 85.21±24.35 | 22.95±10.43 | <0.0001 | 0.413±0.149 | 0.116±0.073 | 0.0002 | 0.395±0.117 | 0.211±0.066 | 0.0017 |
| IVA | 11.27±4.307 | 5.428±3.655 | 0.0111 | 0.023±0.009 | 0.020±0.012 | 0.5286 | 0.110±0.068 | 0.081±0.494 | 0.3499 |
| VA | 14.07±6.462 | 8.779±3.902 | 0.0676 | 0.067±0.025 | 0.043±0.018 | 0.0462 | 0.115±0.088 | 0.047±0.015 | 0.0488 |
| CA | 23.16±10.34 | 11.83±7.593 | 0.0255 | 0.057±0.028 | 0.049±0.021 | 0.5162 | 0.242±0.059 | 0.188±0.039 | 0.0482 |

**Table S5. Quantitative concentrations of SCFAs after *B. lactis*** A6 treatment

|  | **Serum (ng/ml)** | | **P value** | **Muscle (μg/g)** | | **P value** | **Bone (μg/g)** | | **P value** |
| --- | --- | --- | --- | --- | --- | --- | --- | --- | --- |
|  | **DSS** | ***B. lactis* A6** |  | **DSS** | ***B. lactis* A6** |  | **DSS** | ***B. lactis* A6** |  |
| AA | 522.4±175.0 | 625.5±281.3 | 0.3935 | 22.38±6.100 | 30.54±5.286 | 0.0126 | 1.608±0.657 | 2.458±1.041 | 0.0712 |
| PA | 133.8±33.68 | 167.2±28.05 | 0.0490 | 0.326±0.057 | 0.319±0.118 | 0.8804 | 0.305±0.073 | 0.458±0.222 | 0.0857 |
| IBA | 299.6±44.22 | 321.4±68.59 | 0.4635 | 1.565±0.244 | 1.869±0.302 | 0.0439 | 0.077±0.046 | 0.151±0.069 | 0.0238 |
| BA | 22.00±7.353 | 47.89±12.01 | 0.0001 | 0.124±0.052 | 0.278±0.073 | 0.0003 | 0.206±0.074 | 0.046±0.211 | 0.0068 |
| IVA | 5.475±2.495 | 6.544±2.668 | 0.4219 | 0.018±0.006 | 0.024±0.006 | 0.0452 | 0.065±0.047 | 0.075±0.040 | 0.6554 |
| VA | 8.035±2.569 | 11.36±3.449 | 0.0461 | 0.041±0.009 | 0.038±0.010 | 0.5594 | 0.070±0.043 | 0.074±0.060 | 0.8797 |
| CA | 12.81±4.453 | 11.69±5.164 | 0.6477 | 0.050±0.025 | 0.061±0.029 | 0.4324 | 0.175±0.073 | 0.280±0.102 | 0.0333 |
