# Supplementary material for: Bifidobacterium animalis subsp. lactis A6 ameliorates bone and muscle loss via modulating gut microbiota composition and enhancing butyrate production
Source: Bone Res. 2025 Feb 25;13:28. doi: 10.1038/s41413-024-00381-1 (PMC11862215; doi:10.1038/s41413-024-00381-1)
Supplement: Supplementary file 2 — WB-Unprocessed data [file 41413_2024_381_MOESM2_ESM.pdf]

IKB

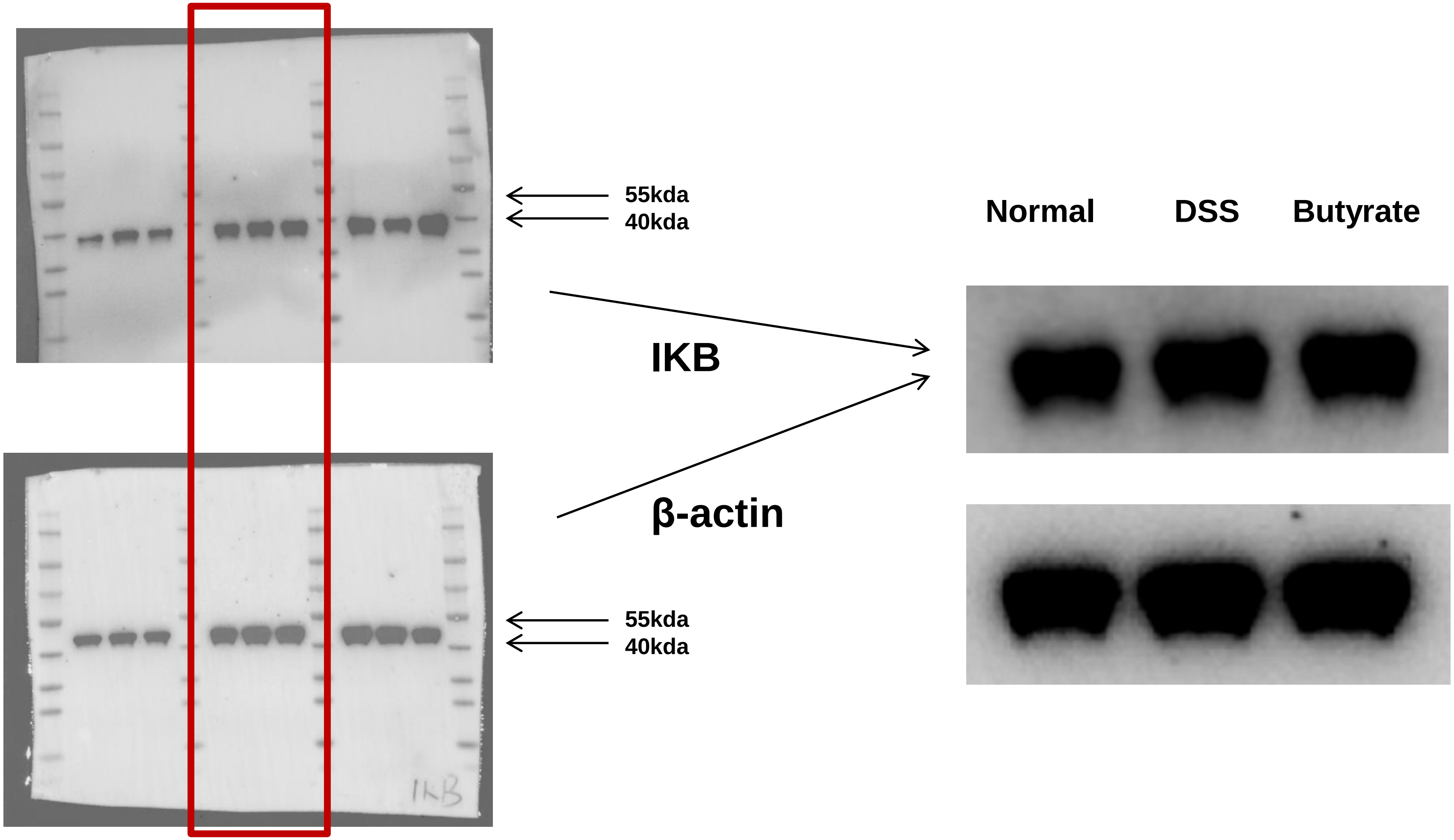

P-IKB

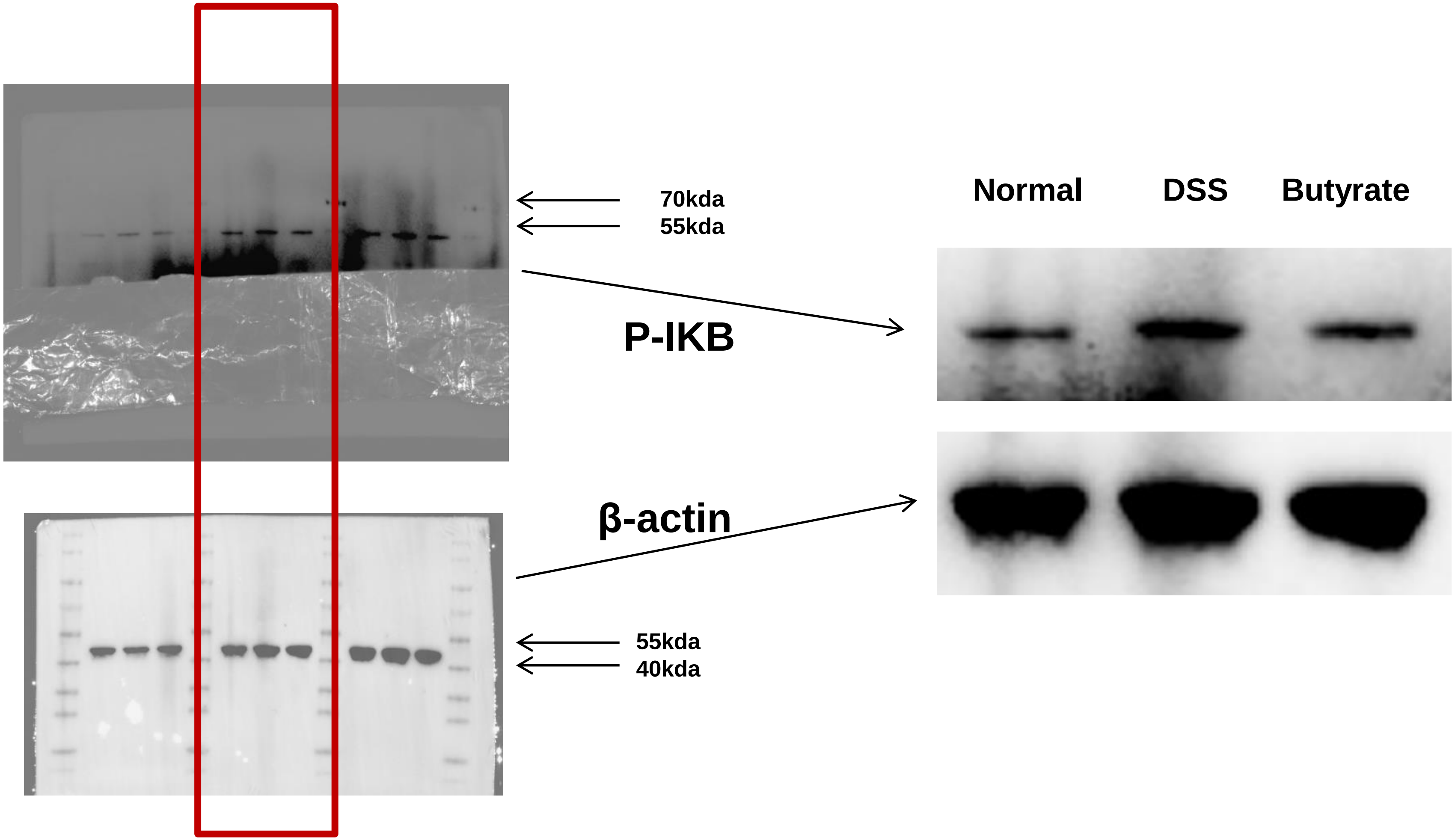

P65

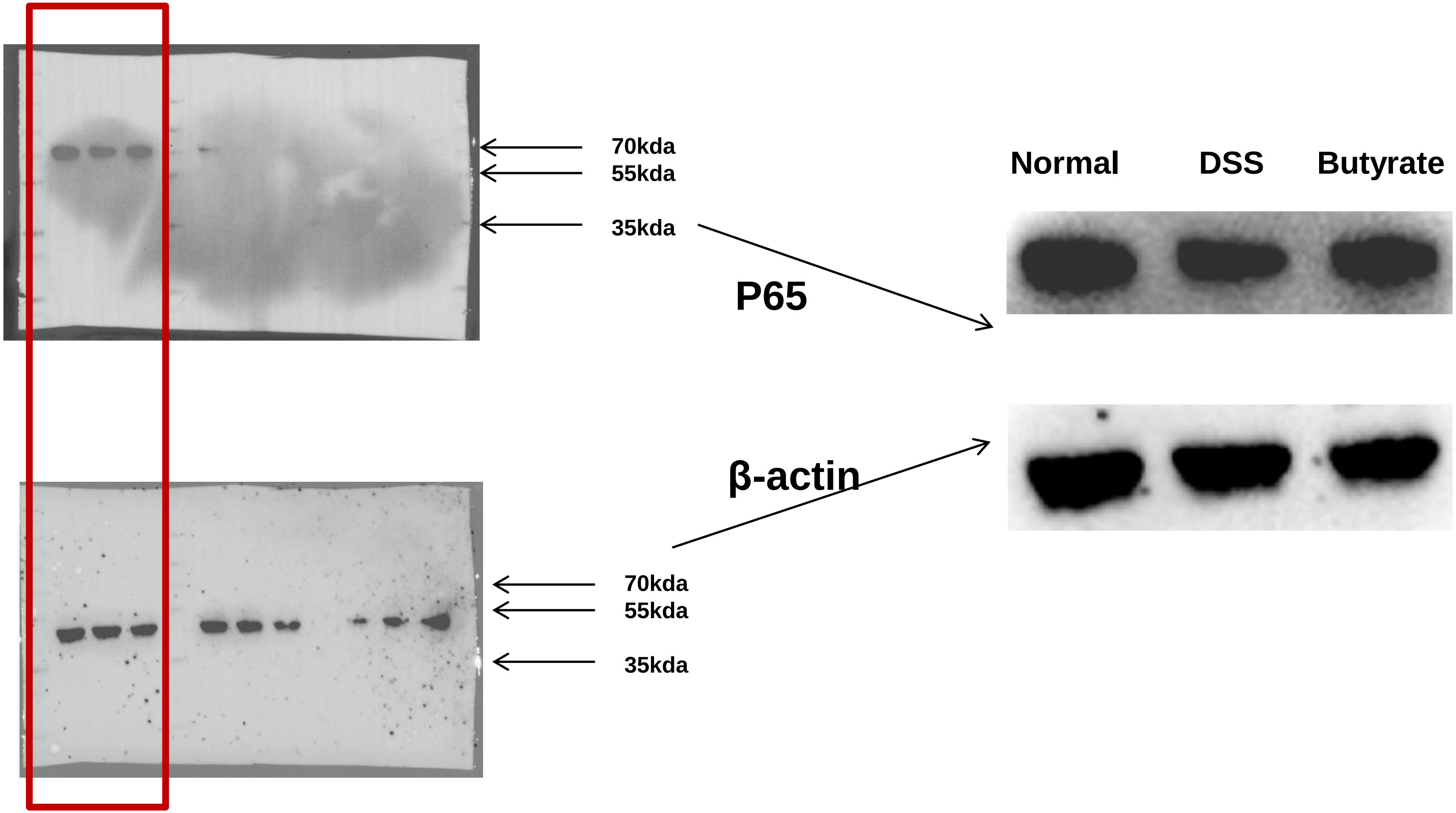
70kda
55kda
35kda
P65
β-actin
70kda
55kda
35kda
Normal
DSS
Butyrate

P-P65

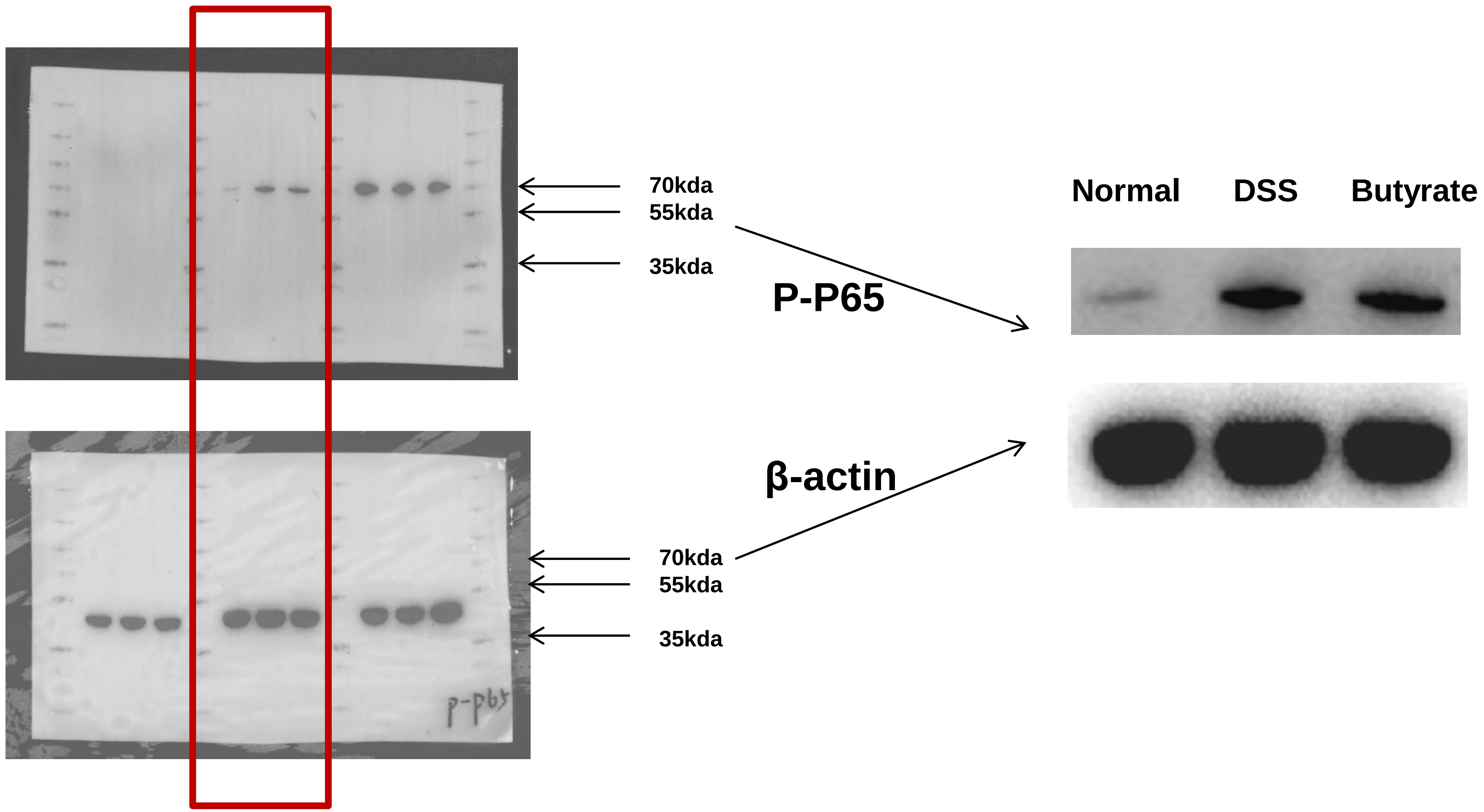
70kda
55kda
35kda
P-P65
β-actin
70kda
55kda
35kda
Normal
DSS
Butyrate
